# Dynamic genome evolution in a model fern

**DOI:** 10.1038/s41477-022-01226-7

**Published:** 2022-09-01

**Authors:** D. Blaine Marchant, Guang Chen, Shengguan Cai, Fei Chen, Peter Schafran, Jerry Jenkins, Shengqiang Shu, Chris Plott, Jenell Webber, John T. Lovell, Guifen He, Laura Sandor, Melissa Williams, Shanmugam Rajasekar, Adam Healey, Kerrie Barry, Yinwen Zhang, Emily Sessa, Rijan R. Dhakal, Paul G. Wolf, Alex Harkess, Fay-Wei Li, Clemens Rössner, Annette Becker, Lydia Gramzow, Dawei Xue, Yuhuan Wu, Tao Tong, Yuanyuan Wang, Fei Dai, Shuijin Hua, Hua Wang, Shengchun Xu, Fei Xu, Honglang Duan, Günter Theißen, Michael R. McKain, Zheng Li, Michael T. W. McKibben, Michael S. Barker, Robert J. Schmitz, Dennis W. Stevenson, Cecilia Zumajo-Cardona, Barbara A. Ambrose, James H. Leebens-Mack, Jane Grimwood, Jeremy Schmutz, Pamela S. Soltis, Douglas E. Soltis, Zhong-Hua Chen

**Affiliations:** 1 Department of Biology, Stanford University, Stanford, CA USA; 2 Central Laboratory, State Key Laboratory for Managing Biotic and Chemical Threats to the Quality and Safety of Agro-products, Zhejiang Academy of Agricultural Sciences, Hangzhou, China; 3 College of Agriculture, Yangtze University, Jingzhou, China; 4 College of Agriculture and Biotechnology, Zhejiang University, Hangzhou, China; 5 School of Science, Western Sydney University, Penrith, New South Wales Australia; 6 College of Life and Environmental Sciences, Hangzhou Normal University, Hangzhou, China; 7 Boyce Thompson Institute, Ithaca, NY USA; 8 Genome Sequencing Center, HudsonAlpha Institute for Biotechnology, Huntsville, AL USA; 9 United States Department of Energy Joint Genome Institute, Lawrence Berkeley National Laboratory, Berkeley, CA USA; 10 Arizona Genomics Institute, School of Plant Sciences, University of Arizona, Tucson, AZ USA; 11 Institute of Bioinformatics, University of Georgia, Athens, GA USA; 12 Department of Biology, University of Florida, Gainesville, FL USA; 13 Department of Biological Sciences, University of Alabama in Huntsville, Huntsville, AL USA; 14 Department of Crop, Soil, and Environmental Sciences, Auburn University, Auburn, AL USA; 15 Plant Biology Section, Cornell University, Ithaca, NY USA; 16 Justus-Liebig-University, Department of Biology and Chemistry, Institute of Botany, Gießen, Germany; 17 Matthias Schleiden Institute/Genetics, Friedrich Schiller University Jena, Jena, Germany; 18 Hubei Insect Resources Utilization and Sustainable Pest Management Key Laboratory, College of Plant Science and Technology, Huazhong Agricultural University, Wuhan, China; 19 Institute of Crops and Nuclear Technology Utilization, Zhejiang Academy of Agricultural Sciences, Hangzhou, China; 20 State Key Laboratory for Managing Biotic and Chemical Threats to the Quality and Safety of Agro-products, Institute of Virology and Biotechnology, Zhejiang Academy of Agricultural Sciences, Hangzhou, China; 21 Institute for Forest Resources & Environment of Guizhou, Key Laboratory of Forest Cultivation in Plateau Mountain of Guizhou Province, College of Forestry, Guizhou University, Guiyang, China; 22 Department of Biological Sciences, University of Alabama, Tuscaloosa, AL USA; 23 Department of Integrative Biology, University of Texas at Austin, Austin, TX USA; 24 Department of Ecology and Evolutionary Biology, University of Arizona, Tucson, AZ USA; 25 Department of Genetics, University of Georgia, Athens, GA USA; 26 New York Botanical Garden, Bronx, NY USA; 27 Department of Plant Biology, University of Georgia, Athens, GA USA; 28 Florida Museum of Natural History, University of Florida, Gainesville, FL USA; 29 Hawkesbury Institute for the Environment, Western Sydney University, Penrith, New South Wales Australia

**Keywords:** Plant evolution, Phylogenetics, Plant sciences

## Abstract

The large size and complexity of most fern genomes have hampered efforts to elucidate fundamental aspects of fern biology and land plant evolution through genome-enabled research. Here we present a chromosomal genome assembly and associated methylome, transcriptome and metabolome analyses for the model fern species *Ceratopteris richardii*. The assembly reveals a history of remarkably dynamic genome evolution including rapid changes in genome content and structure following the most recent whole-genome duplication approximately 60 million years ago. These changes include massive gene loss, rampant tandem duplications and multiple horizontal gene transfers from bacteria, contributing to the diversification of defence-related gene families. The insertion of transposable elements into introns has led to the large size of the *Ceratopteris* genome and to exceptionally long genes relative to other plants. Gene family analyses indicate that genes directing seed development were co-opted from those controlling the development of fern sporangia, providing insights into seed plant evolution. Our findings and annotated genome assembly extend the utility of *Ceratopteris* as a model for investigating and teaching plant biology.

## Abstract (web summary)

The genome of the model fern species *Ceratopteris richardii* reveals a history of remarkably dynamic genome evolution, including rapid changes in genome content and structure following the most recent whole-genome duplication approximately 60 million years ago.

## Main

Ferns have shaped life on Earth since divergence from their common ancestor with seed plants over 360 million years ago (Ma)^1^. Ferns can be found across diverse ecosystems as colonizers^2^, keystone species^3^, invasives^4^ and agricultural supplements^5^, and with over 10,500 extant species, they are the second most species-rich clade of vascular plants behind angiosperms^6^. Accompanying enormous morphological and ecological diversity, ferns have evolved numerous adaptations for protection from environmental stresses^7^. Fern secondary metabolites and their associated genes provide valuable resources for bioremediation, agricultural applications and lifesaving drugs^8–10^.

Ferns have notoriously immense genomes (average 1C, 12.3 billion bases (Gb); maximum 1C, 147 Gb) and very high chromosome numbers (average, 40.5; maximum, 720)^11^, hypothesized to be the consequences of repeated rounds of whole-genome duplication (WGD)^12,13^. However, genetic and genomic signatures of rampant WGD in ferns have not been documented^14–16^. Unfurling the genetic complexities and processes that have shaped fern genomes will illuminate not only the evolutionary history of this phylogenetically pivotal plant clade, but also the evolution of genome features and gene function in seed plants.

**Fig. 1:**
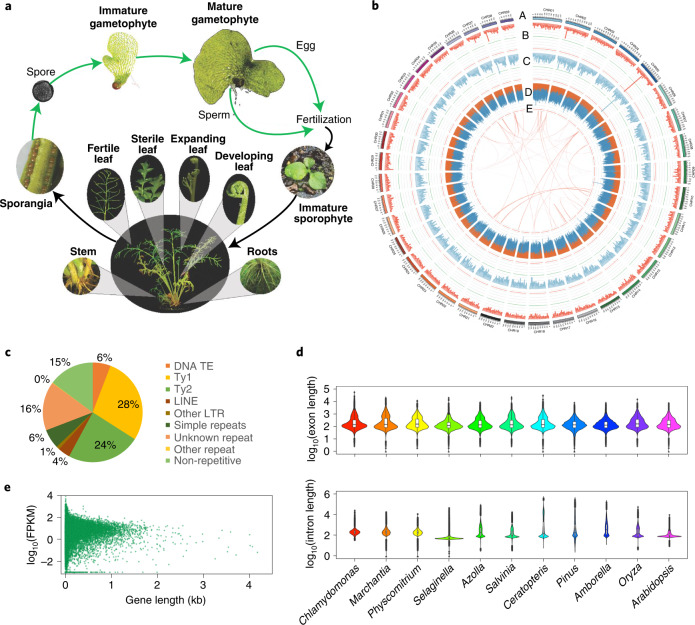
*Ceratopteris richardii* life cycle and genome assembly characteristics. **a**, Life cycle of *Ceratopteris* with tissues sampled for RNA-seq in bold. Images are not to scale. **b**, Genome assembly of *Ceratopteris* with: (A) chromosomes, (B) gene density in a 3-Mb sliding window, maximum value of 139; (C) mRNA expression density in a 3-Mb sliding window, maximum value of 170; (D) long terminal repeat retrotransposon density in a 3-Mb sliding window, orange and blue bands represent Ty3 and Ty1 LTRs, respectively, maximum value of 970; and (E) intragenomic syntenic regions of ten or more genes. Green horizontal lines represent the 5th percentile, red horizontal lines represent the 95th percentile. **c**, Genome composition of *Ceratopteris*. LTR, long terminal repareat; TE, transposable element. **d**, Intron and exon lengths from a green alga (*n* = 166,499 exons; 147,269 introns), liverwort (*n* = 137,019 exons; 112,345 introns), moss (*n* = 587,902 exons; 501,233 introns), lycophyte (*n* = 197,720 exons; 162,895 introns), two water ferns (*Azolla*: *n* = 127,875 exons; 107,674 introns; *Salvinia*: *n* = 122,980 exons; 103,200 introns), *Ceratopteris* (*n* = 437,785 exons; 400928 introns), gymnosperm (*n* = 268,745 exons; 187,344 introns), basal angiosperm (*n* = 111,241 exons; 83,928 introns), monocot (*n* = 196,916 exons; 152,273 introns) and eudicot (*n* = 313,952 exons; 237,746 introns). The widest part of the violin plot represents the highest point density, whereas the top and bottom are the maximum and minimum data respectively. Box plots are in the middle of violin plots, the top and bottom lines represent 25th and 75th percentiles, the centre line is the median and whiskers are the full data range. **e**, Correlation between fragments per kilobase million (FPKM) and gene length at genome-wide level of *Ceratopteris*. Source data

*Ceratopteris richardii* (hereafter *Ceratopteris*) has long been a model for investigating and teaching plant biology (for example, C-Fern Curriculum)^17^. *Ceratopteris* is typical of most ferns in being homosporous (producing a single spore type with potentially bisexual gametophytes) and having a large genome with numerous chromosomes (1C = 9.6 Gb; *n* = 39) relative to most eukaryotes. By contrast, all seed plants (flowering plants and gymnosperms) are heterosporous (producing both male and female spores with unisexual gametophytes). *Ceratopteris* and other homosporous ferns as well as lycophytes also have independent, free-living haploid gametophytes and diploid sporophytes (Fig. 1a), unlike seed plants in which the gametophyte is dependent upon the dominant sporophyte. As such, plant research laboratories globally have incorporated *Ceratopteris* to investigate life history traits, reproductive biology, development, evolution, space biology and genome biology^18,19^. Heterosporous water ferns (Salviniales; <1% of all fern species), characterized by relatively small, compact genomes, are represented by two genome assemblies, *Azolla filiculoides* (1C = 0.75 Gb, *n* = 22) and *Salvinia cucullata* (1C = 0.26 Gb, *n* = 9). These two genomes serve as ideal heterosporous fern counterparts to *Ceratopteris*^20^, but are not representative of the vast majority of ferns.

Here we present the chromosome-level genome assembly and associated genetic resources for *Ceratopteris*. We investigated the composition and evolution of the large genome typical of ferns, analysed DNA methylation, documented horizontal gene transfer (HGT) events, investigated the evolution of gene families essential to flower and seed development, and characterized genes of potential economic, medicinal and environmental importance. The reference genome assembly, annotation and associated datasets extend the utility of *Ceratopteris* as a foundational model species for integration of comparative genomics into plant science research and education.

## Results and discussion

### The impact of transposons on genome size and intron length

We sequenced and assembled 7.46 Gb of the *Ceratopteris richardii* genotype Hn-n genome (https://phytozome-next.jgi.doe.gov/info/Crichardii_v2_1) (Fig. 1b). The *k*-mer analyses yielded a genome size estimate of 9.6 Gb, 15% smaller than previous estimates by flow cytometry^15^ but within the error range of such estimates for large genomes^21,22^. The assembly contains 10,785 contigs with a contig *N50* of 2.3 Mb and scaffold *N50* of 182 Mb, with 93.5% of the assembled sequence contained in the 39 *Ceratopteris* chromosomes (Table 1). This is one of the largest haploid genomes with a chromosomal assembly to date, surpassed only by the assembly of the giant sequoia (*Sequoiadendron giganteum*) genome, which totals 8.125 Gb in 11 chromosomes but with a contig *N50* of 348 kb and scaffold *N50* of 690 Mb^23^.

**Table 1:** Final summary statistics for chromosome-scale assembly

Genome assembly statistics	Number/size
Scaffold total	6,185
Contig total	10,785
Scaffold sequence total	7,463.3 Mb
Chromosome sequence	6,932.2 Mb
Contig sequence total	7,417.3 Mb (0.6% gap)
Scaffold *L*/*N50*	19/182.0 Mb
Contig *L*/*N50*	908/2.3 Mb

Transposable elements vary wildly in number and proportion of the genome among major lineages of life, among related species and even among populations^24^. Long terminal repeat (LTR) retrotransposons (Class I RNA transposable elements), which copy and paste throughout the genome when active, are often the dominant group of repeat elements in plants^25^. Although the transposition of LTR retrotransposons into genes or regulatory regions can disrupt gene function, they can alternatively generate novelty at the genetic, regulatory, expression or isoform levels, providing genetic material for evolution and adaptation^26^.

We found over seven million repetitive elements that account for 85.2% of the *Ceratopteris* assembly (Supplementary Table 1). LTR retrotransposons represented the majority (67.0%) of the genome assembly, with the Ty3 superfamily making up 23.8% of the genome and the Ty1 superfamily making up 28.2%. LTR retrotransposons within these superfamilies averaged 2,301 and 1,492 bp in length, respectively. The Class II DNA transposable elements composed only 6.9% of the genome, with the highest representation from the CMC-En/Spm family, whereas 6.3% of the assembled genome was made up of simple repeat elements (Fig. 1c).

Protein-coding regions were annotated using a combination of ab initio prediction and transcript evidence from isoform sequencing (Iso-Seq) and RNA sequencing (RNA-seq) derived from ten tissues and developmental stages of *Ceratopteris* (Fig. 1a). The annotation of the total genome assembly contains 36,857 protein-coding genes and 38,397 alternative transcripts with 33,567 (91%) protein-coding loci anchored to the 39 chromosomes (Supplementary Table 2). Of the 410 genes in the Viridiplantae (Odb10) Benchmarking Universal Single-Copy Orthologs (BUSCO; v.4.1.1) dataset, 94.8% were identified in the annotation of *Ceratopteris*^27^. The chromosome assembly has, on average, a gene density of 4.81 per Mb, gene length of 14,457 bp, exon length of 363 bp and intron length of 5,555 bp (Supplementary Table 2). Among the extreme outliers, we identified 706 genes in *Ceratopteris* over 100 kb in length. Remarkably, introns account for 30% of the *Ceratopteris* genome with 17,745 introns over 10 kb in length. Although exon length varied little among the major lineages of plants, intron length in *Ceratopteris* had the largest range, beyond even that of the 22-Gb genome of *Pinus taeda*^28^ (Fig. 1d).

Analyses of compact flowering plant genomes, such as *Arabidopsis* and rice, with average intron lengths of 152 and 387 bp, respectively^29^, document a positive correlation between intron length and gene expression^29,30^. However, we found no correlation between total gene length and expression in *Ceratopteris* (*r*^2^ = 0.00004, *P* > 0.05; Fig. 1e). *Ceratopteris* may serve as a model for investigating functional aspects of intron length and content on gene expression and messenger RNA maturation.

### WGD is masked by rapid genome evolution

Polyploidy has contributed to the complexity and gene content of all green plants^31,32^; however, its frequency in the evolutionary history of ferns has been contentious for decades^12,14,33,34^. The large size and numerous chromosomes of fern genomes have long been considered evidence that repeated polyploidy and subsequent gene loss/silencing have contributed to the diversification of molecular and ecological function across fern evolutionary history^34–37^. However, analyses of angiosperm genomes have demonstrated that chromosome number is a poor predictor of WGD frequency^31,38^. Surprisingly, the limited genetic and genomic data now available for ferns have pointed towards polyploidy being less frequent and genome content being less dynamic compared with angiosperms^14–16,39,40^.

**Fig. 2:**
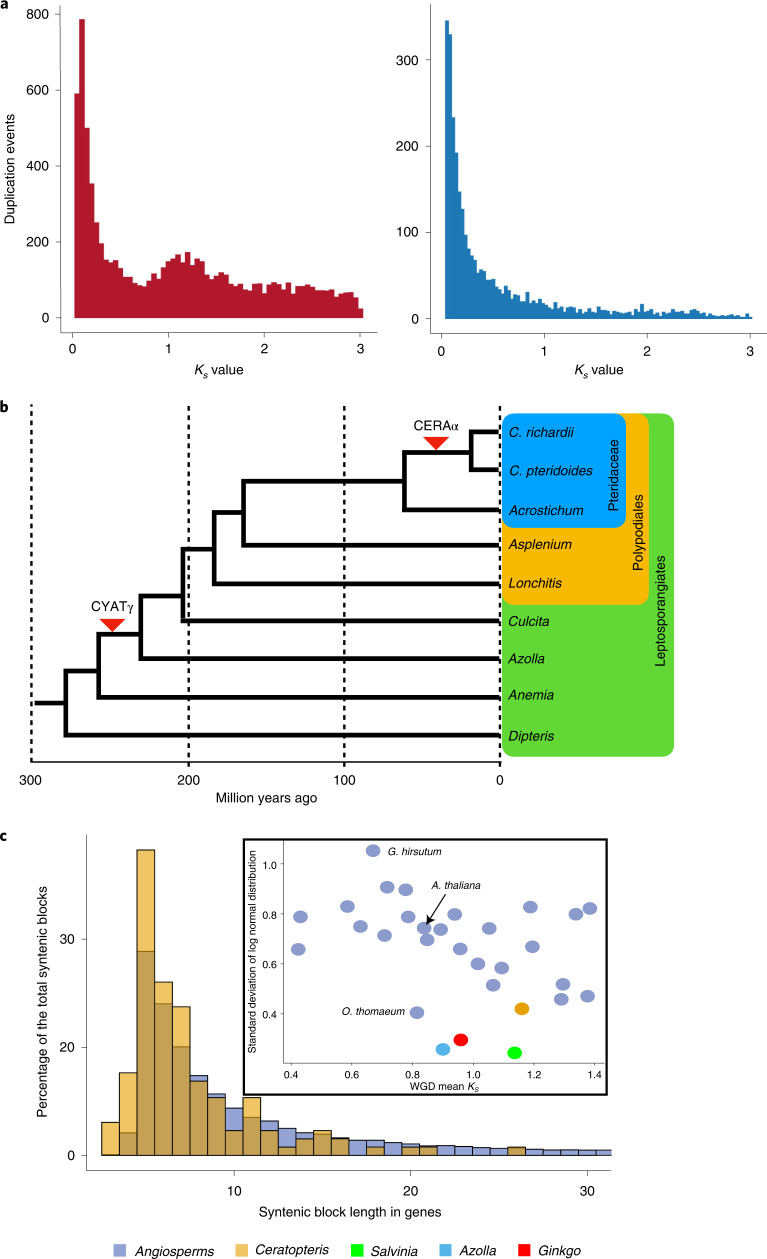
Evidence of polyploidy in the evolutionary history of *Ceratopteris*. **a**, *K*_*s*_ distributions of all paralogous genes (left) and tandemly duplicated paralogous genes (right). **b**, Placement of WGD events in the evolutionary history of *Ceratopteris* based on phylogenomic analyses. **c**, Proportion and length of syntenic regions in *Ceratopteris* (yellow) relative to the average of 27 flowering plant species (lilac). Inset shows the standard deviation of syntenic block length distribution relative to peak *K*_*s*_ value (WGD) for *Ceratopteris* (yellow), *Azolla* (blue), *Salvinia* (green), *Ginkgo* (red) and 27 flowering plant species (lilac). Source data

**Extended Data Fig. 1:**
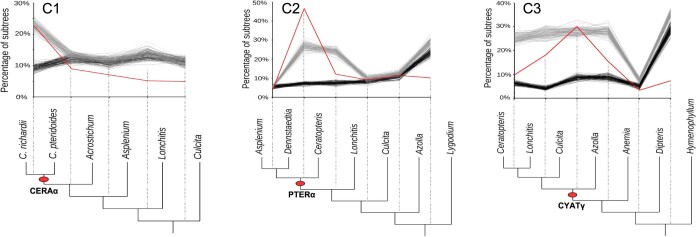
MAPS; observed results and null and positive simulations. Percentage of subtrees that contain a gene duplication shared by descendant species at each node, results from observed data (red line), 100 resampled sets of null simulations (black lines), and positive simulations (gray lines). The red oval corresponds to the putative ancient WGD.

To clarify the impact of WGD on the evolutionary history of *Ceratopteris* and ferns more generally, we employed divergence-based, genomic and phylogenomic approaches. A single WGD event could be inferred from the paralogue synonymous substitution (*K*_*s*_) distribution analysis of *Ceratopteris* with a *K*_*s*_ peak at 1.3 (Fig. 2a). Phylogenetic analyses using Multi-tAxon Paleopolyploidy Search (MAPS)^41^ and NOTUNG^42,43^ of more than 5,000 gene families, including protein sequences from *Ceratopteris* and other fern species, implicated two WGDs on the lineage leading to *Ceratopteris* within the last 300 million years (Myr) as inferred previously^31^ (Fig. 2b). These analyses placed the most recent WGD (CERAα) after the divergence of *Ceratopteris* from its sister genus, *Acrostichum*, at just 62 Ma (ref. ^44^; Fig. 2b and Extended Data Fig. 1), and queries of gene trees indicated that this was the only WGD event represented in the 1.3 peak observed in the *K*_*s*_ plot (Fig. 2a). Both phylogenetic analyses of fern gene sequences also supported a putative earlier WGD before divergence of the Polypodiales and Salviniales lineages 230 Ma (CYATγ), consistent with other fern WGD analyses (Fig. 2b)^45–47^. MAPS also inferred a third ancient WGD in the ancestry of the Polypodiales (PTERα)^31^, but this was not observed in the NOTUNG analyses or another recent analysis using a different set of phylogenomic methods^47^. Additional genomes from the Polypodiales are needed to resolve the ultimate number and position of WGDs in this region of the fern phylogeny.

**Extended Data Fig. 2:**
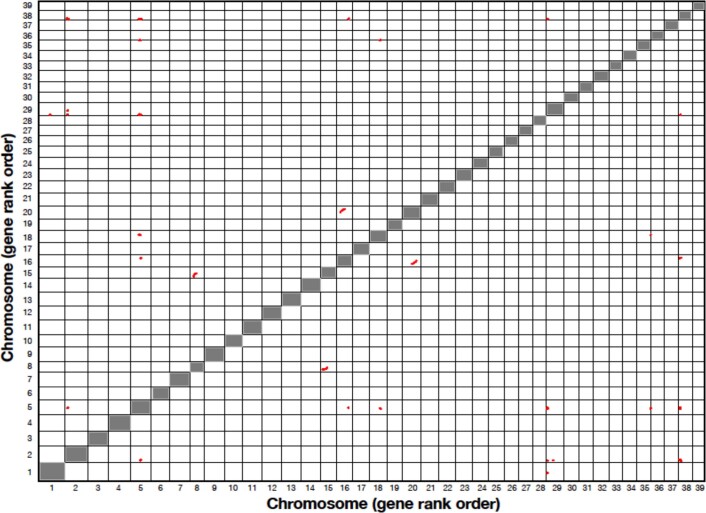
Self-synteny analysis of *Ceratopteris* by chromosome (gene rank order). Syntenic blocks (≥10 genes) are identified in red.

**Extended Data Fig. 3:**
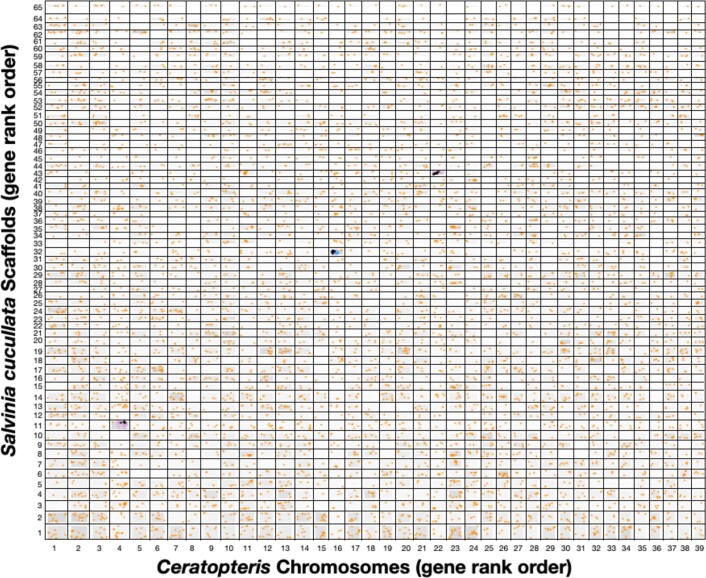
Synteny analysis of *Ceratopteris* chromosomes and *Salvinia cucullata* scaffolds. Syntenic anchor BLAST hits are black points, nearby non-anchor BLAST hits are blue points, and non-syntenic reciprocal best score BLAST hits are orange points.

We expected to find numerous, large syntenic subgenome blocks of paralogous genes among the chromosomes of *Ceratopteris*, the typical genomic signature of WGD. However, only 45 syntenic blocks of ten or more genes, totalling 367 genes, were found within the 39 *Ceratopteris* chromosomes (Extended Data Fig. 2). For comparison, the paralogue *K*_*s*_ distribution for *Arabidopsis thaliana* (1C = 135 Mb, *n* = 5) exhibits median peak values of 0.7, 1.7 and 2.7 for the *At-α*, *At-β* and *At-γ* paleopolyploidy events dated at 23.3 Ma (ref. ^38^) to 50.1 Ma (ref. ^48^), 61.2 Ma (ref. ^48^) and >125 Ma (ref. ^38^) respectively. Syntenic subgenome blocks are evident for all three events, although *At-γ* blocks are highly fragmented relative to syntenic segments of *Arabidopsis* subgenomes attributed to the *At-α* and *At-β* WGDs^49^. Similarly, three independent WGD events (ρ, σ and τ) can be discerned via phylogenetics and synteny in the lineage leading to rice (*Oryza sativa*; 1C = 430 Mb, *n* = 12)^31^. We further tested the scale of retained synteny across fern genomes by comparing the genome of *Ceratopteris* with that of the water fern *Salvinia cucullata*, which last shared a common ancestor 230 Ma (ref. ^20^). Virtually no syntenic blocks were detected between the two fern species (Extended Data Fig. 3).

Diploidization, the process of returning a polyploid genome to a genetically diploid state via fractionation (loss) and silencing of WGD-derived genes, transposition events and genome rearrangements, can vary in rate among plant lineages^50–52^. To assess the degree of synteny in *Ceratopteris* relative to other land plant genomes, we compared syntenic block lengths in *Ceratopteris*, the two sequenced water ferns (*Azolla filiculoides* and *Salvinia cucullata*), a gymnosperm (*Ginkgo biloba*) and 27 angiosperms ranging in WGD history^53^. Only the grass *Oropetium thomaeum* had a smaller average length of syntenic blocks among the angiosperms sampled compared with *Ceratopteris* (Fig. 2c); however, it also has a small genome of 245 Mb and nine chromosomes. All four non-angiosperms had smaller syntenic blocks than the angiosperms other than *O. thomaeum*. These results suggest that although retention of synteny can be highly variable among land plants, the genomes of *Ceratopteris* and the other analysed non-angiosperms are much more fractionated relative to angiosperms. Tandem gene duplications have highly influenced the genome content and structure of *Ceratopteris* because recent tandem duplications account for a large proportion of the paralogue pairs included in the *K*_*s*_ distribution (>6,000 genes) (Fig. 2a). Taken together, we document rapid rates of genome evolution in *Ceratopteris* relative to those of angiosperms that serve to mask WGD events.

### DNA methylation in *Ceratopteris*

**Fig. 3:**
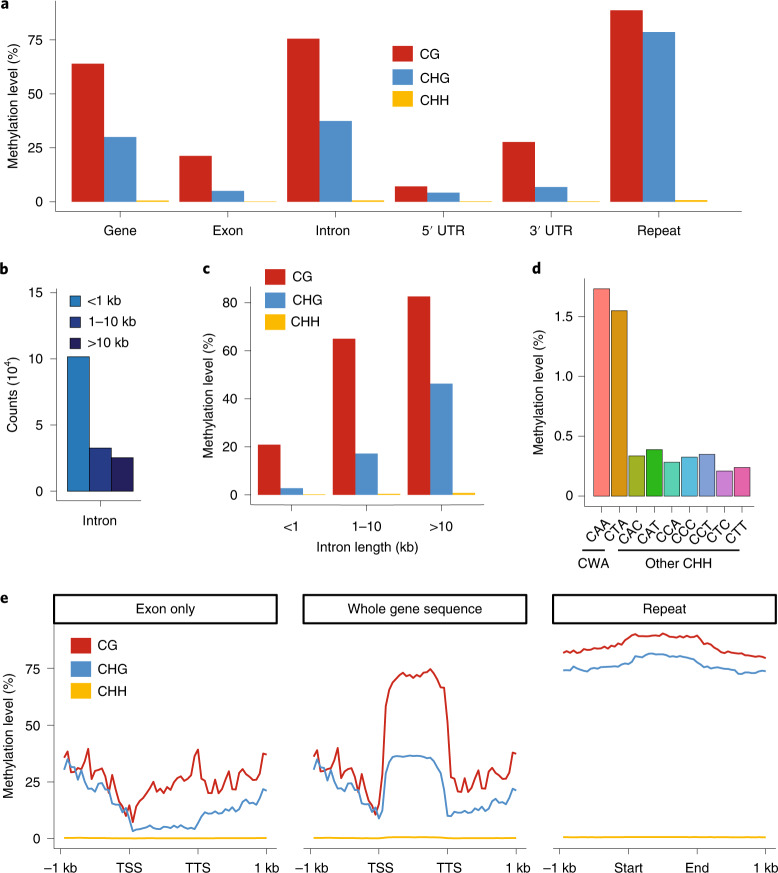
Whole-genome methylation profiling of *Ceratopteris*. **a**, CG, CHG and CHH methylation level in all genes (whole gene sequence) (*n* = 36,857), exons (*n* = 159,326), introns (*n* = 122,469), untranslated region (UTR) (*n* = 32,307) and randomly selected repeats (*n* = 1,000). **b**, Introns were classified into three groups based on length: shorter than 1 kb (*n* = 101,510); 1–10 kb (*n* = 32,547); and longer than 10 kb (*n* = 25,269). **c**, CG, CHG and CHH methylation level for introns of different lengths. **d**, Methylation level of nine CHH contexts in repeats. **e**, Metaplots show distribution of DNA methylation level across gene and repeat body (right). For genes, methylation level was calculated for C-contexts in exons (left) and in whole gene sequences, including exons and introns (middle). TSS, transcription start site. TTS, transcription termination site. Source data

Whole-genome bisulfite sequencing^54,55^ enabled fine-scale resolution of DNA methylation in the *Ceratopteris* genome. CG and CHG methylation (H = A, C or T) were found throughout most genomic features; however, CHG methylation was especially enriched in repeats as well as the unusually large introns (Fig. 3a–e). Interestingly, CHH methylation initially appeared absent in the *Ceratopteris* genome because it could not be readily distinguished from background (Fig. 3a). In angiosperms, CHH methylation results from activities of the RNA-directed DNA methylation pathway and/or CHROMOMETHYLASE 2 (CMT2). In *Arabidopsis*, CMT2 has a preference for CWA (W = A or T) sites^56^. Closer examination of the CHH methylation results revealed CWA sites to be more highly methylated compared with other CHH contexts, albeit at low levels (Fig. 3d). Previous studies have shown that *CMT2* is not present in ferns because it evolved in the common ancestor of angiosperms^57^. However, there are two *CMT* genes present in *Ceratopteris*; at least one of these ancient CMTs presumably possesses the ability to methylate CWA sites. Our data suggest that CMT-associated CWA methylation is present in *Ceratopteris*, but the RNA-directed DNA methylation pathway is not active, consistent with its loss in certain fern species^58^.

Curiously, gene body DNA methylation (gbM), which is common in angiosperms^59^, is also present in *Ceratopteris* (Fig. 3e). gbM is associated with methylation of CG sites only and is present in genes that are often expressed constitutively, evolving slowly and possess ‘housekeeping’ functions^60^. Although it has been hypothesized that gbM could be present outside angiosperms^57,60^, notably within certain gymnosperms and ferns, high coverage genome-wide data were lacking until now to confirm its presence. We therefore provide unambiguous documentation of gbM outside of angiosperms.

### Gene family evolution across green plants

**Fig. 4:**
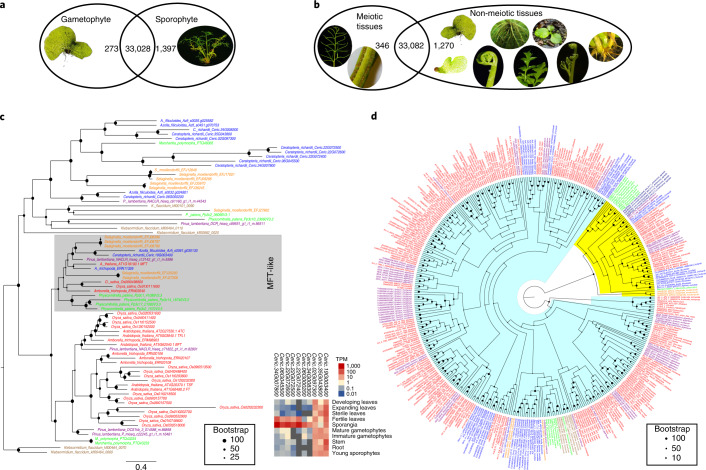
Transcriptome profiling and evolution of gene families for plant reproduction and architecture. **a**, Venn diagram of gametophyte- and sporophyte-specific genes and associated expression heatmaps with log_2_(transcripts per million). **b**, Venn diagram of meiotic- and non-meiotic-specific genes and associated expression heatmaps with log_2_(transcripts per million). **c**, Phylogeny of *FT* genes and their expression in *Ceratopteris*. The MFT-like gene clade is highlighted in grey. **d**, Phylogeny of Type II MADS-box genes across green plants. MIKC*-group genes are highlighted in yellow, MIKC^C^-group genes are highlighted in light blue. For **c** and **d**, angiosperm genes are red, gymnosperm genes are purple, fern genes are blue, lycophyte genes are orange, bryophyte genes are green and algal genes are brown. TPM, transcripts per million. Source data

The identification of genes contributing to reproduction in homosporous ferns and their expression patterns can elucidate the evolution and potential origin of genes driving shifts in the reproductive biology of heterosporous ferns and seed plants^18^. Despite the considerable morphological and physiological differences between the *Ceratopteris* gametophyte and sporophyte (plus the respective haploidy versus diploidy of these alternating generations), only 273 and 1,397 genes were specifically expressed in the gametophyte and sporophyte, respectively (Fig. 4a). Similarly, 346 genes were solely expressed in meiotic tissues (fertile leaf and sporangia), whereas 1,270 genes were solely expressed in non-meiotic tissues and over 30,000 genes were expressed in both datasets (Fig. 4b). This low level of specificity supports recent work suggesting that leaf and seed developmental genes are co-opted from sporangia developmental networks^61–64^.

To better understand the evolutionary transition from seedless plants to the production of seeds, flowers and fruits, we identified and analysed gene families in *Ceratopteris* known to be critical to flower induction in *Arabidopsis* and other angiosperms. The phosphatidyl-ethanolamine binding protein family is well conserved across green plants and animals controlling a variety of biological processes^65^. In angiosperms, the phosphatidyl-ethanolamine binding proteins FLOWERING LOCUS T (FT) and MOTHER OF FT (MFT) regulate flowering time and flower architecture^66^. We identified ten *FT* genes in *Ceratopteris*, compared with six in *Arabidopsis* and four in *Azolla filiculoides* (Fig. 4c). Of those ten, nine *Ceratopteris FT* homologues are present in subfamilies that are absent in flowering plants, whereas the one remaining, and most generally expressed, *Ceratopteris FT* gene was in the clade containing the *Arabidopsis* gene *AtMFT* (Fig. 4c). The three generally expressed *Ceratopteris FT* genes likely play major roles in the many phase changes of the fern life cycle. Interestingly, seven *Ceratopteris FT* homologues were highly expressed only in meiotic tissue (fertile leaf and sporangia), suggesting that these *FT* homologues may be associated with spore development in ferns, predating the function of regulating flowering in angiosperms (Fig. 4c).

### The evolution of plant architecture

MADS-box genes have been identified in almost all eukaryotes, but have expanded most in green plants, where they are well known for their roles in numerous aspects of plant architecture and development^67^. More than 20 years ago, the first MADS-box genes were identified in *Ceratopteris*^68–70^, but owing due to the lack of genomic data, the entire complement of MADS-box genes in a homosporous fern genome has been unclear until now. We identified 35 MADS-box genes in the *Ceratopteris* genome, classified into 8 Type I and 27 Type II MADS-box genes based on phylogeny reconstruction. Type II genes were further subdivided into MIKC^C^- and MIKC*-group genes based on a separate phylogenetic analysis of Type II genes (Fig. 4d). MIKC^C^-group genes are of special interest owing to their crucial importance for flower development and evolution^71^. Studies on *Ceratopteris* in the pre-genomics era had already identified three clades of fern-specific genes (*CRM1-*, *CRM3-* and *CRM6/CRM7-*like genes), with each clade containing several paralogues^68,72^. Twenty-one Type II genes belong to the clade of MIKC^C^-group genes, and six are MIKC*-group genes. Surprisingly, recent analyses based on comparative transcriptomics additionally identified a large ‘orphan’ clade of previously unknown fern-specific MIKC^C^-group genes for which no *Ceratopteris* representative was previously known^31^. Analysis of the *Ceratopteris* genome corroborates the view that there are no orphan clade members in this species. Because representatives exist in all major groups of ferns^31^, these genes must have been established early in fern evolution and lost relatively recently in the lineage that led to *Ceratopteris*. Interestingly, even though MIKC*-group genes have more exons (9–12) than MIKC^C^-group genes (6–8 exons), all of the genomic loci of MIKC*-group genes were smaller (<40 kb) than all of the genomic loci of MIKC^C^-group genes. Nine MIKC^C^-type genes are encoded by genomic loci spanning 100,000 to 216,247 bp. The first intron, known to include regulatory elements in other plants^73–76^, is often the largest intron of *Ceratopteris* MADS-box genes. Notably, we also found two MIKC^C^-group MADS-box genes each encompassing two alternative MADS boxes. For one of these genes, we found mRNAs including one or the other, but not both MADS boxes. For the other gene, unfortunately, there is not enough mRNA data to judge which mRNAs are formed. The generation of mRNAs from these loci with multiple MADS boxes potentially involves alternative promoters and differential splicing. A similar phenomenon has so far only been described for a number of MADS-box genes in Norway spruce, *Picea abies*^77^.

### HGT and the evolution of defence genes

**Extended Data Fig. 4:**
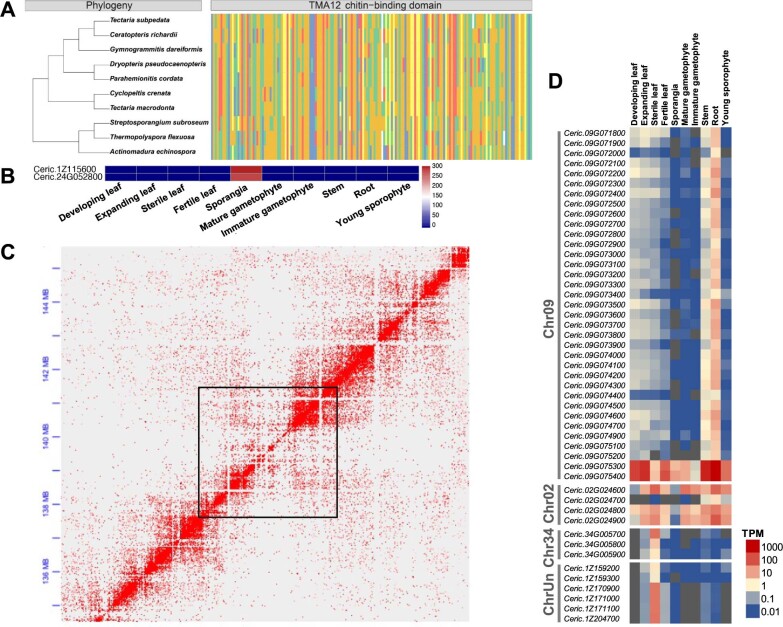
Horizontal gene transfers (HGTs) and their evolution among ferns. **A**, Phylogeny of *Tma12* with protein conservation analysis of the chitin-binding domain. **B**, expression heatmap in tissues of *Ceratopteris*. **C**, HiC anchoring of Chromosome 9 with the region where the aerolysin-like genes are located outlined in black, confirming their presence in the *Ceratopteris* genome assembly. **D**, Expression patterns of the aerolysin-like genes in *Ceratopteris*. Source data

Novel biopesticides have been discovered in a number of fern species and have benefited sustainable agriculture and food security^10,78^. For example, a gene encoding a novel insecticidal protein, *Tma12*, was identified in the fern *Tectaria macrodonta* and cloned into cotton to battle phytophagous whiteflies^10^. *Tma12* was also identified in the genome of *Salvinia cucullata* and in the transcriptomes of other ferns^20^. Phylogenetic placement of the fern *Tma12* genes among bacterial sequences suggests that the fern genes originated from HGT from bacteria to ferns^20^ (Extended Data Fig. 4a). We identified two homologues of *Tma12* in *Ceratopteris*, expression of which differed dramatically between tissues and developmental stages, with sporangia showing over 3,000 times greater expression compared with the rest of the RNA-seq samples (Extended Data Fig. 4b). Similar to increased defensive metabolite production in unripe fruits^79^, *Tma12* expression in *Ceratopteris* may be an adaptation that protects the sporangia from insect attack before spore dispersal.

**Fig. 5:**
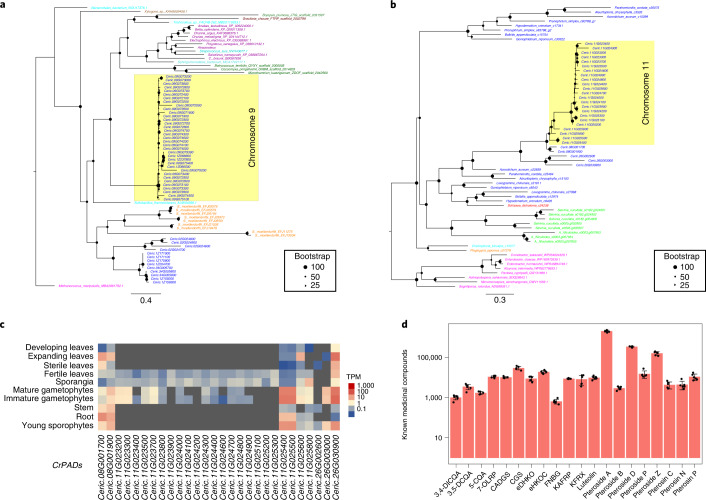
HGTs and medicinal compounds in *Ceratopteris*. **a**, Phylogeny of aerolysin-like genes suggests an HGT from bacteria to early vascular plants and a second HGT specifically to ferns, followed by tandem duplications on chromosome 9 in *Ceratopteris* (highlighted). Fern genes are blue, lycophyte genes are orange, fish are purple, bacteria are cyan, fungi are brown, archaea are magenta, chlorophytic algae are dark green and red algae are rust. **b**, Phylogeny of *PAD* genes in *Ceratopteris* and leptosporangiate ferns suggests an HGT from bacteria to ferns followed by rampant tandem duplication across chromosome 11 (highlighted). Polypodiales are blue, Schizaeales are red, Salviniales are light green, Gleicheniales are cyan, Cyatheales are orange and bacteria are pink. **c**, Expression of *Ceratopteris* PAD genes (*CrPADS*) across tissues/life stages. **d**, Metabolic profile of previously identified medicinal compounds in *Ceratopteris*. CADGS, casuarine 6-alpha-d-glucoside; CGS, casuarine 3-glucoside; 5-CQA, *cis*-5-caffeoylquinic acid; 3,5-DCQA, 3,5-di-*O*-caffeoylquinic acid; 3,4-DICQA, 4,5-di-*O*-caffeoylquinic acid; eDHKO, ent-7alpha,12beta-dihydroxy-16-kauren-19,6beta-olide; eHKOC, ent-17-hydroxy-15-kauren-19-oic acid; FNBG, flavanone_7-*O*-beta-d-glucoside; KAFRP, kaempferol 3-arabinofuranoside 7-rhamnofuranoside; KFRX, kaempferol 3-rhamnoside 7-xyloside; luteolin, 2,4′,5,7-tetrahydroxyflavanone; 7-OLRP, 7-*O*-alpha-l-rhamnopyranoside. Data are presented as means ± s.e.m. (*n* = 6). Source data

**Extended Data Fig. 5:**
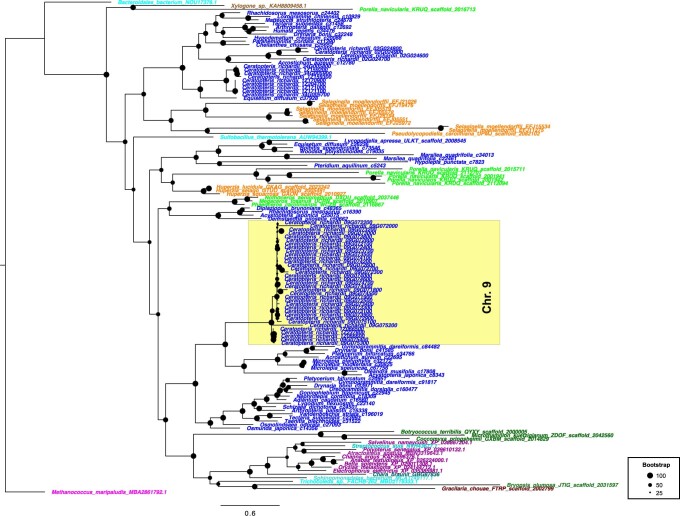
Transcriptome-based phylogeny of aerolysin-like genes. Two horizontal gene transfers are evident, one fern-specific and one in early land plants. Aerolysin-like genes that were tandemly duplicated on Chromosome 9 are highlighted. Fern genes are blue, lycophyte genes are orange, bryophyte genes are light green, fish genes are purple, bacteria genes are cyan, fungal genes are brown, archaea genes are magenta, streptophytic algae genes are black, chlorophytic algae genes are dark green, and red algae genes are rust.

We discovered a block of 36 recently tandemly duplicated aerolysin-like protein-coding genes on chromosome 9 of *Ceratopteris* (Fig. 5a). These genes are well-studied in bacteria because they encode a pore-forming cytolytic toxin that forms channels in plasma membranes and has been widely incorporated into biological nanopore research^80,81^. Aerolysin-like genes are hypothesized to have recurrently undergone HGT among different kingdoms^82^. Searches of plant transcriptomic datasets^31,83^ found aerolysin-like transcripts in the transcriptomes of each major lineage of land plants with the exception of seed plants (Extended Data Fig. 5). However, when we confined our search to gene models from reference genome assemblies, aerolysin-like genes were found only in *Ceratopteris* and the lycophyte *Selaginella moellendorffii*, as well as diverse algae, fungi, bacteria and fish. HiC data anchored the large array of aerolysin-like genes on chromosome 9 (Extended Data Fig. 4c), refuting the possibility of contamination in *Ceratopteris*. Four and three additional copies of aerolysin-like genes were found on chromosomes 2 and 34, respectively. We found subfunctionalization of the aerolysin-like genes among the different tissues of *Ceratopteris*; 34 of the aerolysin-like genes on chromosome 9 were highly expressed in the stem and roots, and all three of the aerolysin-like genes on chromosome 34 were highly expressed in sterile leaves. One aerolysin-like gene on chromosome 2 was highly expressed in stem, roots and young sporophytes, and the remaining aerolysin-like genes were generally expressed across all tissues (Extended Data Fig. 4d). Similar to *Tma12*, these aerolysin-like genes are likely the result of HGT, possibly recurrent, from bacteria to early land plants. We expect future non-angiosperm reference genomes will provide support for the presence of these genes in plants beyond *Ceratopteris* and *Selaginella* and clarify their evolutionary history.

A second block of tandemly duplicated genes was found on chromosome 11. These genes were annotated as phenolic acid decarboxylases (PADs), which are of particular interest because they catalyse the non-oxidative decarboxylation of potentially toxic phenolic acids to their *p*-vinyl derivatives^84^. PADs are of distinct interest to bioengineering because they have been proposed as biocatalysts given that these vinyl derivative compounds can be used as polymer precursors and flavour/fragrance additives in the food-processing industry^84^. To date, *PAD* genes have only been documented in bacteria; however, we discovered 26 *PAD* genes in *Ceratopteris*, 21 of which originated from tandem duplications on chromosome 11 (43–45 cM). We further searched plant transcriptomic datasets for *PADs* and found them solely in leptosporangiate ferns with bacteria as the sister clade (Fig. 5b). Similar to the aerolysin-like genes, the *PADs* have subfunctionalized because 20 genes were highly expressed in fertile leaves, sporangia and gametophytes, whereas the remaining six were generally expressed across all tissues and developmental stages (Fig. 5c). HGT coupled with rapid diversification via tandem duplications and subfunctionalization of these genes in *Ceratopteris* together provide unique insight into land plant evolution and the integration of novel genes.

### The medicinal potential of fern genomics

Ferns have long been used in traditional medicine worldwide and more recently have been a source for bioprospecting medicinal compounds to treat cancer, diabetes and osteoarthritis^9,85–87^. However, the lack of a high-quality reference genome of a homosporous fern species has hindered the research and development of novel fern compounds for medicinal application. Here we leveraged the genomic resources from *Ceratopteris* and performed metabolite profiling of sporophytic tissue to investigate potentially medicinal compounds produced by *Ceratopteris* and the genes underlying their production.

Metabolite profiling of *Ceratopteris* fertile leaf tissue identified several known medicinal compounds, including eight pterosides, seven flavonoids, three caffeic acids and two kauranes (Fig. 5d and Supplementary Table 3). We identified 906 high-confidence metabolites in *Ceratopteris*, compared with 644 metabolites in wheat and rice^88^ (Supplementary Table 3). Of those 906 metabolites, 57 were unique compounds only detected in *Ceratopteris* and 131 were novel because they could not be annotated using known metabolome databases.

**Extended Data Fig. 6:**
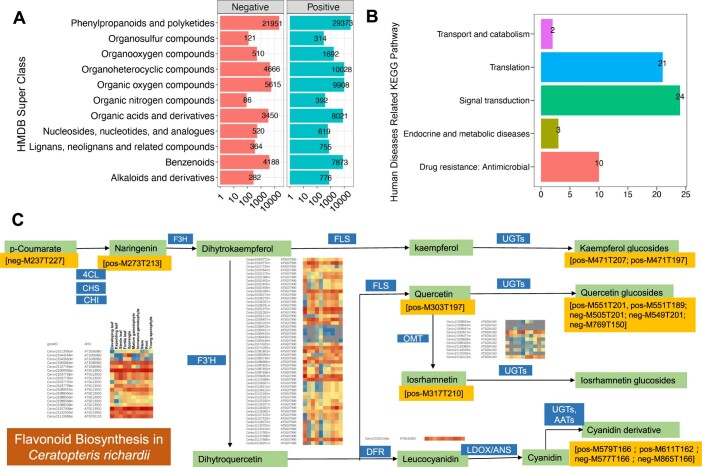
Analysis of *Ceratopteris* genes and metabolites for applications in environment and medicine. The metabolites identified in all 6 biological replications were used in this study. **A**, Overview of the metabolomics dataset of *Ceratopteris* based on HMDB (Human Metabolome Database, https://hmdb.ca). All compounds were divided into 11 clades according to the HMDB superclass. The compound numbers in each superclass were separated as positive and negative metabolites. **B**, KEGG enrichment of putative compounds related to human diseases. **C**, The Flavonoid Biosynthesis pathway in *Ceratopteris*. 4CL, 4-coumarate:CoA ligase; CHS, chalcone synthase; CHI, chalcone isomerase; F3H, flavanone 3-hydroxylase; F3’H, flavonoid 3’-hydroxylase; FLS, flavonol synthase; OMT1, O-methyltransferase; UGT, UDPdependent glycosyltransferase; DFR, dihydroflavonol 4-reductase; LDOX/ANS, leucoanthocyanidin dioxygenase/anthocyanidin synthase; AAT, anthocyanin acyltransferase. Source data

High-confidence compounds related to the treatment of human diseases were identified in *Ceratopteris*, with enrichment in pathways such as flavonoid biosynthesis, endocrine and metabolic disease, and antimicrobial function (Extended Data Fig. 6). Flavonoids are vital for human nutrition, healthcare and medicine^89^. The flavonoid biosynthesis pathway is specific to land plants and arose when plants colonized land over 470 Ma (ref. ^90,91^). In angiosperms, flavonoids play crucial roles in abiotic stress tolerance and are also important for pollination and seed dispersal signalling^91^. Our combined analyses identified the genes, expression patterns and metabolites that compose the flavonoid biosynthesis pathway in *Ceratopteris* (Extended Data Fig. 6c). Alongside such metabolic analyses, our high-quality *Ceratopteris* genome will readily advance our understanding of the molecular origins and functions of these known and novel compounds to benefit drug discovery in ferns to improve human health.

## Conclusions

Homosporous ferns have been the last frontier in green plant genomics owing to their notoriously large genomes and numerous chromosomes; the mechanisms driving the evolution of these genomes have been debated for decades. Despite longstanding hypotheses of rampant WGD in ferns, the *Ceratopteris* genome assembly revealed evidence for at least two WGD events distributed over 300 Myr of fern evolution. By contrast, the *Arabidopsis* and rice genomes each exhibit evidence for three independent sets of WGD events over roughly 125 Myr of flowering plant evolution^31^. Surprisingly, syntenic genomic segments were not evident for even the most recent WGD in *Ceratopteris* owing to frequent tandem duplications, high rates of fractionation and genome rearrangements. Defence-related gene families expanded via extensive tandem duplications and probably originated from separate HGTs from bacteria. In addition, we document CMT-associated CWA methylation and provide unambiguous evidence of gbM in a fern. Importantly, we traced the evolution of genes involved in flower and seed development and overall plant architecture to homologues in fern genes. Ferns have been underutilized as sources of novel genetic material for applications in ecological remediation, medicine and bioprospecting; we demonstrate the potential of these resources. Beyond these scientific discoveries, *Ceratopteris* has long been utilized in biology classrooms as a model for teaching the alternation of generations in green plants^17,92^. With the genetic, genomic and metabolomic resources provided in this study, *Ceratopteris* can become the primary plant system for teaching next-generation plant biology. In conclusion, the *Ceratopteris* genome data provide critical resources for future investigations of gene function in this fern model, and support research in plant biology, genome evolution, biotechnology and medicine, as well as advancing plant biology curricula.

## Methods

### Genome sequencing

We sequenced *Ceratopteris richardii* genotype Hn-n using a range of sequencing technologies, including single-molecule real-time long-read sequencing from Pacific Biosciences (PacBio), chromosome conformation capture using HiC sequencing, Illumina short-read sequencing, bisulfite sequencing, PacBio Iso-Seq and RNA-seq (Supplementary Table 4). High molecular mass DNA was isolated from fresh leaf tissue at the Arizona Genomics Institute. Sequencing reads were collected using Illumina and PacBio platforms. Illumina and PacBio reads were sequenced at the Department of Energy Joint Genome Institute in Walnut Creek, CA, USA and the HudsonAlpha Institute for Biotechnology in Huntsville, AL, USA. Illumina reads were sequenced using the Illumina NovaSeq platform, and the PacBio reads were sequenced using the SEQUEL platform. Before assembly, Illumina fragment reads were screened for PhiX contamination. Reads composed of >95% simple sequences were removed. Illumina reads <50 bp after trimming for adaptor and quality (q < 20) were removed. The final read set consists of 2,438,428,350 reads for a total of 47.27× of high-quality Illumina bases. For the PacBio sequencing, a total of 93 PB chemistry 2.1 chips (10-h movie time) was sequenced with a sequence yield of 777.1 Gb, with a total coverage of 69.02× (Supplementary Table 5).

### RNA-seq

Total RNA was isolated from ten tissues and developmental stages of *Ceratopteris richardii* genotype Hn-n (Fig. 1a) using the RNeasy Plant Mini kit (Qiagen). Plate-based RNA sample preparation was performed on the PerkinElmer Sciclone NGS robotic liquid handling system using Illumina’s TruSeq Stranded mRNA HT sample prep kit utilizing poly-A selection of mRNA following the protocol outlined by Illumina in their user guide (https://support.illumina.com/sequencing/sequencing_kits/truseq-stranded-mrna.html) with the following conditions: total RNA starting material was 1 µg per sample and eight cycles of PCR were used for library amplification. There are four biological replicates for each tissue and developmental stage of the RNA-seq experiment.

### Iso-Seq

With 1 µg of total RNA as input, full-length complementary DNA was synthesized using template switching technology with the SMARTer PCR cDNA Synthesis kit (Clontech). The first-strand cDNA was amplified with PrimeSTAR GL DNA polymerase (Clontech) using template switching oligos to make double-stranded cDNA. Double-stranded cDNA was purified with non-size selected AMPure PB beads (PacBio). The amplified cDNA was end-repaired and ligated with blunt-end PacBio sequencing adaptors using SMRTbell Template Prep Kit 1.0. The ligated products were treated by exonuclease to remove unligated products and purified by AMPure PB beads (PacBio).

### Genome assembly and construction of pseudomolecule chromosomes

The v.2.0 assembly was generated by error correcting the 52,299,716 PacBio reads (69.02× sequence coverage) using MECAT assembler v.1.1 (ref. ^93^), followed by assembly with the Canu assembler v.1.8 (ref. ^94^) and subsequent polishing using ARROW^95^. This produced an initial assembly of 35,249 contigs, with a contig *N50* of 1.5 Mb, 11,081 scaffolds larger than 100 kb and a total assembled size of 9,204.7 Mb (Supplementary Table 6).

Misjoins in the assembly were identified using HiC data as part of the JUICER pipeline^96^. A total of 44 misjoins was identified in the polished assembly. The contigs were then oriented, ordered and joined together using HiC data. In all, 5,031 joins were applied to the broken assembly to form the final assembly of 39 chromosomes. Each chromosome join is padded with 10,000 Ns (placeholders representing any base). A substantial amount of telomeric sequence was identified using the (TTAGGG)_*n*_ repeat, and care was taken to make sure that contigs terminating in telomeres were properly oriented in the production assembly. The remaining scaffolds were screened against bacterial proteins, organelle sequences and GenBank nr (non-redundant proteins) and removed if found to be a contaminant. Additional scaffolds were classified as repetitive (>95% masked with 24mers that occur more than four times in the genome) (21 scaffolds, 784.5 kb), prokaryote (158 scaffolds, 8.4 Mb), low quality (composed of <70% PACBIO polished bases) (5 scaffolds, 26.3 kb), redundant (409 scaffolds, 15.2 Mb) and contaminant (361 scaffolds, 4.8 Mb).

Chromosomal scaffolding and validation were enabled with HiC sequencing of the Hn-n genotype and genome skimming of the 58 double haploid lines derived from a HαPQ45 (paraquat-tolerant mutant of Hn-n) × ΦN8 cross^15^. Homozygous single nucleotide polymorphisms (SNPs) and insertions/deletions (INDELs) were corrected in the release consensus sequence using 42× of Illumina reads (2 × 150, 400 bp insert) by aligning the reads using bwa mem^97^ and identifying homozygous SNPs and INDELs with GATK’s UnifiedGenotyper tool^98^. A total of 676 homozygous SNPs and 13,913 homozygous INDELs were corrected in the release. The final v.2.0 release contains 7,417.3 Mb of sequence, consisting of 10,785 contigs with a contig *N50* of 2.3 Mb and a total of 93.5% of assembled bases in the 39 chromosomes.

Over 371,000 transcript assemblies were made from 1.5 billion pairs of 2 × 150 stranded paired-end Illumina RNA-seq reads using PERTRAN, which conducts genome-guided transcriptome short-read assembly via GSNAP^99^ and builds splice alignment graphs after alignment validation, realignment and correction on transcript assemblies from PASA^100^. About 15 million PacBio Iso-Seq circular consensus sequences were corrected and collapsed by a genome-guided correction pipeline to obtain >819,000 putative full-length transcripts. Loci were determined by transcript assembly alignments and/or EXONERATE alignments of proteins from *Arabidopsis thaliana*, *Glycine max*, *Sorghum bicolor, Oryza sativa*, *Setaria viridis*, *Solanum lycopersicum*, *Aquilegia coerulea*, *Vitis vinifera*, *Marchantia polymorpha*, *Spaghnum magellanicum*, *Ceratodon purpureus*, *Selaginella moellendorffii*, *Physcomitrium patens, Nymphaea colorata*, *Amborella trichopoda*, *Papaver somniferum*, *Azolla filiculoides, Salvinia cucullata*, and Swiss-Prot proteomes to repeat-soft-mask the *Ceratopteris richardii* genome using RepeatMasker^101^ with up to 2,000 bp extension on both ends unless extending into another locus on the same strand. A repeat library consisting of de novo repeats by RepeatModeler^102^ and repeats in RepBase was constructed. Gene models were predicted by homology-based predictors, FGENESH+^103^, FGENESH_EST (similar to FGENESH+, but using expressed sequence tags, ESTs, to compute splice sites and intron input instead of protein/translated open reading frames, ORFs), EXONERATE^104^ and PASA assembly ORFs (in-house homology constrained ORF finder). The best-scored predictions for each locus were selected using multiple positive factors including EST and protein support, and one negative factor, overlap with repeats. The selected gene predictions were improved by PASA. Improvement included adding untranslated regions, splicing correction and adding alternative transcripts. PASA-improved gene model proteins were subject to protein similarity analysis to obtain Cscore and protein coverage. Cscore is a protein BLASTP score ratio to mutual best hit BLASTP score, and protein coverage is the highest percentage of protein aligned to the best homologue. PASA-improved transcripts were selected based on Cscore, protein coverage, EST coverage and its coding sequence (CDS) overlapping with repeats. A transcript was selected if its Cscore was ≥0.5 and protein coverage was ≥0.5, or it had EST coverage, but its CDS overlapping with repeats was <20%. For a gene model whose CDS overlaps with repeats for more than 20%, its Cscore must be at least 0.9 and homology coverage at least 70% to be selected. The selected gene models were subject to Pfam analysis, and gene models whose proteins were >30% in Pfam transposable element domains were removed. Incomplete gene models, gene models with low homology support without full transcriptome support and gene models based on short single exons (<300 bp CDS) without protein domain or good expression were manually filtered out.

Completeness of the euchromatic portion of the assembly was assessed using 39,078 annotated genes from the v.1.0 release of *Ceratopteris*. The aim of this analysis is to obtain a measure of completeness of the assembly, rather than a comprehensive examination of gene space. The transcripts were aligned to the assembly using BLAT^105^, and alignments with ≥95% base pair identity and ≥95% coverage were retained. The screened alignments indicate that 38,458 (98.40%) of the previously annotated *Ceratopteris* genes aligned to the v.2.0 release. Of the remaining annotated genes, 126 (0.32%) aligned at <50% coverage and 68 (0.19%) were not found in the v.2.0 release. The predicted proteome showed that there are 37,263 putative proteins and 3,841 predicted signal peptides in *Ceratopteris* (Supplementary Table 7).

### Transcriptome analysis

Clean reads of different transcriptome tissues were mapped to the genome reference by HISAT2 v.2.2.1 (ref. ^106^), then sorted by samtools (v.1.11)^107^. Stringtie v.2.1.4 (ref. ^108^) and the R package ballgown v.2.20.0 (ref. ^109^) were used in quantification of each tissue based on the bam file generated by HISAT2. Tissue/developmental stage-specific genes were those that were only expressed in a single tissue/developmental stage. For example, the 273 gametophyte-specific genes identified in Fig. 4a were expressed in either the mature or immature gametophyte RNA-seq data, but not at all expressed in any of the sporophyte RNA-seq samples. The clusterProfiler package v.3.16.1 (ref. ^110^) in R was used to enrich cluster analysis and the Vennerable v.3.0 package (https://R-Forge.R-project.org/projects/vennerable/) was used in Venn diagram analysis. The heatmaps of transcripts per million in different tissues were generated by the R package pheatmap v.1.0.12 (https://CRAN.R-project.org/package=pheatmap).

### Comparative genomics

Syntenic orthologous and homeologous regions were defined with the GENESPACE pipeline (https://code.jgi.doe.gov/plant/genespace-r)^111^. In short, GENESPACE accomplishes synteny-constrained orthology inference across multiples species, permitting variable ploidy by parsing protein similarity scores into syntenic blocks with MCScanX^112^ and runs Orthofinder^113^ on synteny-constrained BLAST results. The resulting block coordinates and syntenic orthogroups give high-confidence anchors for evolutionary inference. We used default parameters for intergenomic comparisons and very relaxed thresholds for within-*Ceratopteris* WGD tests (no constraint to orthogroups, minimum block size, 5; maximum gaps, 25).

### *K*_*s*_ distributions

*K*_*s*_ distribution analysis was implemented using the wgd (https://github.com/arzwa/wgd) pipeline^114^. Briefly, all-versus-all BLASTp^115^ was used for similarity searches, and the results were then clustered in paralogous families by MCL^116^. Estimation of *K*_*s*_ for all pairs of paralogous genes and tandemly duplicated paralogous genes was performed using the codeml program in the PAML^117^ package with the F3X4 model. In further analyses, we used only gene pairs with *K*_*s*_ values in the range of 0.05–3.0. Histograms of *K*_*s*_ distributions were generated by the ggplot2 package v.3.3.3 (ref. ^118^) in R.

### MAPS and NOTUNG analyses

To determine the phylogenetic placement of ancient WGD events detected in the *Ceratopteris* genome, we used the *Ceratopteris richardii* genome and other fern transcriptomes for MAPS and NOTUNG analyses. For MAPS, orthologous groups for the selected species were obtained from Orthofinder^113^ with the default parameters and only retained gene families that contained at least one gene copy from each taxon. The phylogenetic trees for gene families constructed by PASTA^119^ were analysed by MAPS. Both null and positive simulations of the background gene birth and death rates were performed to compare with the observed number of duplications at each node. For null simulations, we estimated the gene birth rate (*λ*) and death rate (*μ*) for the selected species with WGDgc^120^. Gene count data of each gene family for all species were obtained from Orthofinder^113^. The species tree for each MAPS analysis was obtained based on previous phylogenetic analyses^31^. The estimated parameters *λ* and *μ* were configured in MAPS, and the gene trees were then simulated within the species tree using the ‘GuestTreeGen’ program from GenPhyloData^121^. For each species tree, we simulated 3,000 gene trees with at least one tip per species: 1,000 gene trees at the estimated *λ* and *μ*, 1,000 gene trees at half of the estimated *λ* and *μ*, and 1,000 trees at three times *λ* and *μ*^31,122^. We then randomly resampled 1,000 trees without replacement from the total pool of gene trees 100 times to provide a measure of uncertainty on the percentage of subtrees at each node. A Fisher’s exact test was used to identify locations with significant increases in gene duplication compared with a null simulation. For positive simulations, we simulated gene trees using the same methods described above. However, we incorporated WGDs at the location in the MAPS phylogeny with significantly larger numbers of gene duplications compared with the null simulation. We allowed at least 20% of the genes to be retained following the simulated WGD^31,122^.

We also performed gene tree reconciliation using NOTUNG v.2.9.1.5 (ref. ^123^) with a model of gene duplication and loss without HGTs. We used protein alignments from PASTA^119^ as described above. RAxML v.8.2.11 (ref. ^124^) was used to generate gene family phylogenies with 100 bootstraps. The species trees were rerooted with an outgroup under the ‘-reroot’ function in NOTUNG. The 80% bootstrap value was used as a threshold to rearrange branches with low support on gene trees based on the species tree topology under the ‘-rearrange’ function. The gene tree reconciliation was performed using all gene family phylogenies. The cost of loss was set to 0.1 to account for missing data of transcriptomes^125^.

### Paleologue identification using Frackify

We used Frackify to identify paleologues in the *Ceratopteris* genome originating from the WGD peak at *K*_*s*_ 1.3 (ref. ^53^). Frackify is a machine learning approach that uses multiple features from gene age distributions and synteny to identify paleologues in genomes^53^. Syntenic blocks were identified using MCScanX^112^ set to a minimum match size (-s) of three based on Zhoa and Shranz^126^. This analysis recovered 193 syntenic blocks representing 923 collinear genes. Given that *Ceratopteris* does not have a closely related outgroup with a high-quality reference genome available, we used a version of Frackify trained without an outgroup (https://gitlab.com/barker-lab/frackify). For this analysis, Frackify identified a total of 395 paleologues within the syntenic blocks identified by MCScanX.

To assess the relative rate of fractionation in the *Ceratopteris* genome compared with other plant genomes, we compared the distribution of syntenic block sizes and WGD age for *Ceratopteris, Azolla filiculoides, Salvinia cucullata, Ginkgo biloba* and 27 flowering plant genomes (Supplementary Table 8)^53^. We used MCScanX set to minimum match size (-s) of three to identify synteny blocks in the 31 plant species^126^. We then compared the distribution of syntenic block lengths in *Ceratopteris* against the entire dataset (Fig. 2c). Finally, we used the fitdistr() function from the MASS R library to fit a log normal distribution to the distribution of syntenic block lengths in each species^127^. We compared the standard deviation of the log normal distribution in each species against the mean *K*_*s*_ of the focal WGD (Fig. 2c).

### Whole-genome bisulfite sequencing and DNA methylation patterns on genes and repeats

The *Ceratopteris* bisulfite sequencing data used in this study were processed by Methylpy v.1.4.2 as described in Schultz et al.^128^. Quality filtering and adaptor trimming were performed using cutadapt v.1.18 (ref. ^129^). Qualified reads were aligned to the *Ceratopteris* v.2.1 reference genome using bowtie v.2.4.1 (ref. ^130^). Only uniquely aligned and non-clonal reads were retained (Supplementary Table 9). Lambda genomic DNA was used as a control to calculate the sodium bisulfite reaction conversion rate of unmodified cytosines, which was >99.9% for this sample. A binomial test was used to determine the methylation status of cytosines with a minimum coverage of three reads.

The gene or repeat body was divided into 20 windows. Additionally, regions 1,000 bp upstream and downstream were each divided into twenty 50 bp windows. Methylation levels were calculated for each window according to previous recommendations^128^. The mean methylation level for each window was then calculated for all genes and all repeats, respectively. Locations of genes and repeats were obtained from the annotated gff files of the *Ceratopteris* v.2.0 reference.

The two *CMT* genes (*Ceric.34G031800*, *Ceric.12G007000*) were identified from our annotations (PFAM, PANTHER, KEGG) and possess the BAH, CHROMO and C-5 cytosine-specific DNA methylase domains.

### Evolutionary analysis of gene families

Unless noted otherwise, comparative genetic similarity analysis of gene families across the major green plant lineages and algae was described in Chen et al.^131^. Briefly, the candidate protein sequence was searched in the *Arabidopsis* protein sequences database with the criteria of E-value <10^−5^ and paired with the *Arabidopsis* protein in the first hit (with highest BLAST score). The number and protein similarity of gene families in each plant species were calculated using the *Arabidopsis* gene family as a reference. Genome sequence data of all species were obtained from the National Center for Biotechnology Information (NCBI) (http://www.ncbi.nlm.nih.gov) and Ensembl Plants (http://plants.ensembl.org/index.html). Transcriptomic sequence data of plants were obtained from the One Thousand Plant Transcriptomes (1KP) database^31^ and a recent fern transcriptome analysis^83^. The sequences were aligned with MAFFT^132^, the best model was estimated with IQ-Tree, and the phylogeny was constructed with 1,000 fast-bootstrap replicates^133^.

### MADS-box identification and phylogeny

To identify MADS-box genes in *Ceratopteris*, we first searched for MADS domains in the predicted proteins using HMMER 3.1b2 with a customized hidden Markov model for the MADS domain^134^. To also detect MADS-box genes that may have escaped automatic annotation, the *Ceratopteris* genome was translated in all six possible reading frames into amino acid sequences, and HMMER 3.1b2 searches were repeated on the translated genome. Genomic regions for which new MADS domains were recognized were extracted, including 100,000 nucleotides up- and downstream of the sequence potentially encoding a MADS domain. Genes were predicted on these genomic regions using AUGUSTUS 3.3.2 (ref. ^135^) with the parameters ‘--strand=forward--codingseq=on --species=arabidopsis’.

BLAST searches^136^ of the identified MADS-domain proteins were conducted on NCBI using ‘non-redundant’ protein sequences as the database. In cases where the BLAST results indicated suboptimal gene models, an improved similarity-based gene prediction was attempted using FGENESH+^137^ with the most similar protein as a guide. In some cases, the similarity-based gene prediction also failed, presumably due to the presence of large introns. In these cases, we manually annotated the MADS-box genes. To do so, we compared the genomic regions with the most similar MADS-domain protein using BLAST with the option ‘Align two or more sequences’ and then annotated exons complying with conventional splice sites and keeping an open reading frame. The plausibility of the manually annotated MADS-box genes was checked by searching these sequences in short-read archive data^138^ for *Ceratopteris* transcriptomes on NCBI. We initially identified 39 MADS-box genes in *Ceratopteris* (Supplementary Table 10). Four of these were classified as potential pseudogenes, whereas 16 of the 35 potential genuine MADS-box genes correspond to previously identified MADS-box genes in *Ceratopteris* (Supplementary Table 10)^68–70,139^.

Protein sequences of MADS-domain proteins were aligned using Probalign^140^ on the CIPRES Science Gateway v.3.3 (ref. ^141^). Alignment of the complete set of MADS-domain proteins was trimmed using trimAl 1.2rev59t^142^ with the parameters ‘-gt .9 -st .00001’ to remove positions with low conservation. For the alignment of the Type II MADS-domain proteins, we used the parameters ‘-gt .8 -st .0001’ for the first trimming. Both trimmed alignments were then trimmed with the parameters ‘-seqoverlap 75 -resoverlap 0.7’ to remove sequences with low conservation. Phylogenies were reconstructed using RAxML v.8.2.12 (ref. ^124^) on the CIPRES Science Gateway^141^. Based on the phylogeny of the complete set of MADS-domain proteins, Type I and Type II MADS-domain proteins were separated, and a phylogeny of Type II MADS-domain proteins was reconstructed.

### Identification of *Tma12* in ferns

We used BLASTp to search for *Tma12* (JQ438776 in GenBank) homologues in 1KP transcriptomes^31^ and 69 fern transcriptomes^83^, as well as the published water fern genomes of *Azolla filiculoides* and *Salvinia cucullata*^20^, using an E-value <10^−10^. HMMER 3.1b2 was also used in conserved domain building based on the fungal *Tma12* orthologue sequences and predicted protein searching. After removing the alternative isoforms and filtering the sequences in which the length is shorter than one-third of the reference sequence length, we found six *Tma12* orthologous genes in the fern transcriptomes database, and two in *Ceratopteris* (Ceric.24G052800, Ceric.1Z115600) that have the same amino acid sequences but no hits were found in the 1KP database. In *Salvinia cucullata*, the only hit was found in the genome data, but not in annotated protein sequences. To explore the phylogeny of *Tma12* in ferns, we selected three fungal *Tma12* sequences (*Streptosporangium subroseum*, *Thermopolyspora flexuosa*, *Actinomadura echinospora*) as the outgroup. The protein sequences were aligned with MAFFT^132^, the best model was estimated with IQ-Tree, and the phylogeny was constructed with 1,000 fast-bootstrap replicates^133^.

### Identification of aerolysin-like genes and evolution

We initially identified the aerolysin-like genes on chromosome 9 while investigating blocks of tandemly duplicated genes based on our Panther annotations (PTHR34007). We used BLASTp to search for aerolysin-like homologues for other fern species in 69 fern transcriptomes^83^, for algae in 1KP transcriptomes^31^ and for bacteria in NCBI using an E-value <10^−5^. We then restricted our search to the *Ceratopteris* genome and published genomes as described in the gene family analysis section. The sequences were aligned with MAFFT^132^, the best model was estimated with IQ-Tree and the phylogeny was constructed with 1,000 fast-bootstrap replicates^133^.

### Identification of PADs genes and evolution

We used BLASTp to search for PAD-like homologues in 69 fern transcriptomes^83^ and published genomes (species used in the gene family analysis section) using an E-value <10^−5^. The sequences were aligned with MAFFT^132^, the best model was estimated with IQ-Tree, and the phylogeny was constructed with 1,000 fast-bootstrap replicates^133^.

### Analysis of metabolites

Young sporophytes of *Ceratopteris* were collected with six replicates, and metabolites were extracted with 1:1 methanol:water buffer. The samples were stored at −80°C before liquid chromatography–mass spectrometry analysis. Pooled quality control (QC) samples were also prepared by combining 10 μl of each extract. All samples were analysed using a TripleTOF 5600 Plus high-resolution tandem mass spectrometer (SCIEX) with both positive and negative ion modes. Chromatographic separation was performed using an ultra-performance liquid chromatography system (SCIEX). An ACQUITY UPLC T3 column (100 mm × 2.1 mm, 1.8 µm) was used for the reversed-phase separation.

The TripleTOF 5600 Plus system was used to detect metabolites eluted from the column. The ion spray floating voltages were set at 5 and −4.5 kV for the positive ion mode and negative ion mode, respectively. The mass spectrometry data were acquired in the IDA mode. The TOF mass range was 60–1,200 Da. A QC sample was analysed every ten samples to evaluate the stability of the liquid chromatography–mass spectrometry.

Raw data files were converted into mzXML format and then processed using the XCMS, CAMERA and metaX toolbox in R. Each ion was identified by the comprehensive information of retention time and *m*/*z*. The open access databases, KEGG^143^ and HMDB^144^, were used to annotate the metabolites by matching the exact molecular mass data (*m*/*z*) to those from the database within a threshold of 10 ppm. The peak intensity data were further preprocessed using metaX. Features that were detected in <50% of QC samples or 80% of test samples were removed, and values for missing peaks were extrapolated with the *k*‐nearest neighbour algorithm to improve the data quality further. Data normalization was performed on all samples using the probabilistic quotient normalization algorithm. Then, QC-robust spline batch correction was performed using the QC samples. *P* values from Student’s *t*‐tests were adjusted for multiple tests using an FDR correction (Benjamini–Hochberg) for the metabolite selection. The VIP cut‐off value of 1.0 was set to select important features (Supplementary Table 11).

### Reporting summary

Further information on research design is available in the Nature Research Reporting Summary linked to this article.

## Supplementary information

Supplementary Information Supplementary Discussion, Figs. 1–5 and Table titles 1–12.

Reporting Summary

Supplementary Tables Supplementary Table 1: Repeat annotation statistics of the *Ceratopteris richardii* genome assembly. Supplementary Table 2: Summary statistics of the *Ceratopteris richardii* chromosome assemblies. Supplementary Table 3: Classification of the detected metabolites in *Ceratopteris richardii* compared with wheat and rice. Supplementary Table 4: Sequencing libraries included in the *Ceratopteris richardii* (Hn-n) genome assembly and their respective assembled sequence coverage levels in the final release. Supplementary Table 5: PacBio library statistics for the libraries included in the *Ceratopteris* genome assembly and their respective assembled sequence coverage levels. Supplementary Table 6: Summary statistics of the initial output of the ARROW polished MECAT assembly. Supplementary Table 7: Summary statistics of the *Ceratopteris* predicted proteome. Supplementary Table 8: Angiosperm sampling for Frackify analysis. Supplementary Table 9: Whole-genome bisulfite sequencing statistics of the *Ceratopteris* genome. Supplementary Table 10: MADS-box gene locations and annotation in *Ceratopteris*. Supplementary Table 11: Key metabolites (alkaloids, flavonoids and steroids) in sporophytes of *Ceratopteris*. Supplementary Table 12: CYP450 diversity across green plants (taxa included in the CYP450 phylogeny are highlighted).

## Data Availability

Raw genomic sequences and assemblies have been deposited in the NCBI SRA under BioProject PRJNA729743. Genome and transcriptome assemblies and annotations can be found in Phytozome (https://phytozome-next.jgi.doe.gov/info/Crichardii_v2_1). Publicly available data were collected from Ensembl Plants (plants.ensembl.org), NCBI (ncbi.nlm.nih.gov), Swiss-Prot (uniprot.org), RepBase (girinst.org/repbase), One Thousand Plant Transcriptomes (1KP) database (OTPT Initiative, 2019), fern transcriptome database^83^, water fern genomes (fernbase.org), spruce genome (congenie.org), TAIR (arabidopsis.org). Source data are provided with this paper.

## Source data

Source Data Fig. 1 Statistical source data for Fig. 1d,e.
Source Data Fig. 2 Statistical source data for Fig. 2b,c.
Source Data Fig. 3 Statistical source data for Fig. 3a–e.
Source Data Fig. 4 Statistical source data for Fig. 4c.
Source Data Fig. 5 Statistical source data for Fig. 5c,d.
Source Data Extended Data Fig. 4 Statistical source data for Extended Fig. 4b,d.
Source Data Extended Data Fig. 6 Statistical source data for Extended Fig. 6c.

## Code availability

All custom codes are available for research purposes from the corresponding authors upon request.
