# Supplementary material for: Dynamic genome evolution in a model fern
Source: Nat Plants. 2022 Sep 1;8(9):1038–51. doi: 10.1038/s41477-022-01226-7 (PMC9477723; doi:10.1038/s41477-022-01226-7)
Supplement: Supplementary file 1 — Supplementary Discussion, Figs. 1–5 and Table titles 1–12. [file 41477_2022_1226_MOESM1_ESM.pdf]

nature portfolio

https://doi.org/10.1038/s41477-022-01226-7

Supplementary information

Dynamic genome evolution in a model fern

In the format provided by the authors and unedited

Dynamic genome evolution in a model fern

D. Blaine Marchant[1†*], Guang Chen[2,3†], Shengguan Cai[4,5†], Fei Chen[6], Peter Schafran[7], Jerry Jenkins[8], Shengqiang Shu[9], Chris Plott[8], Jenell Webber[8], John T. Lovell[8,9], Guifen He[9], Laura Sandor[9], Melissa Williams[8], Shanmugam Rajasekar[10], Adam Healey[8], Kerrie Barry[9], Yinwen Zhang[11], Emily Sessa[12], Rijan R. Dhakal[13], Paul G. Wolf[13], Alex Harkess[8,14], Fay-Wei Li[7,15], Clemens Rössner[16], Annette Becker[16], Lydia Gramzow[17], Dawei Xue[6], Yuhuan Wu[6], Tao Tong[3], Yuanyuan Wang[18], Fei Dai[4], Shuijin Hua[19], Hua Wang[20], Shengchun Xu[4], Fei Xu[4], Honglang Duan[21], Günter Theißen[17], Michael R. McKain[22], Zheng Li[23], Michael T.W. McKibben[24], Michael S Barker[24], Robert J. Schmitz[25], Dennis Stevenson[26], Cecilia Zumajo-Cardona[26], Barbara A. Ambrose[26], James H. Leebens-Mack[27*], Jane Grimwood[8*], Jeremy Schmutz[8,9*], Pamela S. Soltis[28*], Douglas E. Soltis[12,28*], Zhong-Hua Chen[5,29*]

[1]Department of Biology, Stanford University, Stanford, CA, 94305, USA
[2]Central Laboratory, State Key Laboratory for Managing Biotic and Chemical Threats to the Quality and Safety of Agro-products, Zhejiang Academy of Agricultural Sciences, Hangzhou, 310021, China
[3]College of Agriculture, Yangtze University, Jingzhou, 434025, China
[4]College of Agriculture and Biotechnology, Zhejiang University, Hangzhou 310058, China
[5]School of Science, Western Sydney University, Penrith, NSW 2751, Australia
[6]College of Life and Environmental Sciences, Hangzhou Normal University, Hangzhou, China
[7]Boyce Thompson Institute, Ithaca, NY 14853, USA
[8]Genome Sequencing Center, HudsonAlpha Institute for Biotechnology, Huntsville, AL 35806, USA
[9]United States Department of Energy Joint Genome Institute, Lawrence Berkeley National Laboratory, Berkeley, CA 94720, USA
[10]Arizona Genomics Institute, School of Plant Sciences, University of Arizona, Tucson, AZ 85721, USA
[11]Institute of Bioinformatics, University of Georgia, Athens, GA 30602, USA
[12]Department of Biology, University of Florida, Gainesville, FL 32611, USA
[13]Department of Biological Sciences, University of Alabama in Huntsville, Huntsville, AL 35899, USA
[14]Department of Crop, Soil, and Environmental Sciences, Auburn University, Auburn, AL 36849, USA
[15]Plant Biology Section, Cornell University, Ithaca, NY 14853, USA
[16]Justus-Liebig-University, Department of Biology and Chemistry, Institute of Botany, Heinrich-Buff-Ring 38, 35392 Gießen, Germany
[17]Matthias Schleiden Institute/Genetics, Friedrich Schiller University Jena, Philosophenweg 12, 07743 Jena, Germany
[18]Hubei Insect Resources Utilization and Sustainable Pest Management Key Laboratory, College of Plant Science and Technology, Huazhong Agricultural University, Wuhan, 430070, China
[19]Institute of Crops and Nuclear Technology Utilization, Zhejiang Academy of Agricultural Sciences, Hangzhou, China
[20]State Key Laboratory for Managing Biotic and Chemical Threats to the Quality and Safety of Agro-products, Institute of Virology and Biotechnology, Zhejiang Academy of Agricultural Sciences, Hangzhou 310021, China
[21]Institute for Forest Resources & Environment of Guizhou, Key Laboratory of Forest Cultivation in Plateau Mountain of Guizhou Province, College of Forestry, Guizhou University, Guiyang 550025, China
[22]Department of Biological Sciences, University of Alabama, Tuscaloosa, AL 35487, USA
[23]Department of Integrative Biology, University of Texas at Austin, Austin, TX 78712, USA
[24]Department of Ecology and Evolutionary Biology, University of Arizona, Tucson, AZ 85721, USA
[25]Department of Genetics, University of Georgia, Athens, GA 30602, USA
[26]New York Botanical Garden, Bronx, NY 10458, USA
[27]Department of Plant Biology, University of Georgia, Athens, GA 30602, USA
[28]Florida Museum of Natural History, University of Florida, Gainesville, FL 32611, USA
[29]Hawkesbury Institute for the Environment, Western Sydney University, Penrith, NSW, Australia

SUPPLEMENTARY INFORMATION

- **Supplementary Discussion**
  - ***Abiotic stress response gene repertoire in ferns***
  - ***Evolution of the* APETALA2 *gene lineage***
  - ***Co-evolution of LEU- and SEU-like gene families***
  - ***Evolution and selection of cell cycle gene families***
  - ***Cytochrome P450 gene family diversity across green plants***
  - ***Revitalizing the C-Fern Curriculum***
- **Supplementary Figure 1.** Phylogeny of *NRAMP* metal-ion transporter gene family and *Ceratopteris* NRAMP gene expression
- **Supplementary Figure 2.** Phylogeny of the *APETALA2/ETHYLENE RESPONSIVE FACTOR* (*AP2/ERF*) across land plants and expression in *Ceratopteris*
- **Supplementary Figure 3.** Co-evolution of transcriptional regulators
- **Supplementary Figure 4.** Expansion and contraction in copy number of cell cycle-related genes across green plants
- **Supplementary Figure 5.** CYP450 diversity across green plants

- **Supplementary Table 1.** Repeat annotation statistics of the *Ceratopteris richardii* genome assembly
- **Supplementary Table 2.** Summary statistics of the *Ceratopteris richardii* chromosome assemblies
- **Supplementary Table 3.** Classification of the detected metabolites in *Ceratopteris richardii* compared with wheat and rice
- **Supplementary Table 4.** Sequencing libraries included in the *Ceratopteris richardii* (Hn-n) genome assembly and their respective assembled sequence coverage levels in the final release
- **Supplementary Table 5.** PacBio library statistics for the libraries included in the *Ceratopteris* genome assembly and their respective assembled sequence coverage levels
- **Supplementary Table 6.** Summary statistics of the initial output of the ARROW polished MECAT assembly
- **Supplementary Table 7.** Summary statistics of the *Ceratopteris* predicted proteome
- **Supplementary Table 8.** Angiosperm sampling for Frackify analysis
- **Supplementary Table 9.** Whole genome bisulfite sequencing statistics of the *Ceratopteris* genome
- **Supplementary Table 10**. MADS-box gene locations and annotation in *Ceratopteris*
- **Supplementary Table 11.** Key metabolites (alkaloids, flavonoids and steroids) in sporophytes of *Ceratopteris*
- **Supplementary Table 12.** CYP450 diversity across green plants (taxa included in the CYP450 phylogeny are highlighted)

SUPPLEMENTARY DISCUSSION

Abiotic stress response gene repertoire in ferns

Ferns are known for their tolerance of heavy metals and metalloids in both soil and freshwater[1–4]. Another *Ceratopteris* species, *C. pteridoides*, accumulates cadmium and is a viable candidate for phytoextraction and remediation of cadmium-contaminated ecosystems[5]. Although the genes directly controlling cadmium accumulation have not been identified, we discovered the expansion and functional diversification of the Natural Resistance-Associated Macrophage Protein (NRAMP) heavy metal transporter gene family, which has been shown to control cadmium uptake in *Arabidopsis* (Supplementary Fig. 7)[6]. Further analyses of heavy metal and metalloid tolerance in various fern species alongside genome-informed experimental investigations of gene function in *Ceratopteris* will expand understanding of molecular processes contributing to heavy metal hyperaccumulation in plants. With their abundant natural defenses and phytoremediation potential, ferns provide an important resource for genetic engineering of crop resistance and environmental restoration.

Evolution of the **APETALA2** *gene lineage*

The *APETALA2/ETHYLENE RESPONSIVE FACTOR* (*AP2/ERF*) family is essential to plant development across green plants and encompasses genes known to control flower, seed, and fruit development in angiosperms[7]. To identify the *APETALA2* and *AINTEGUMENTA* homologs in *Ceratopteris richardii*, we performed a BLAST search using homologs previously reported across land plants[8,9]. A total of 199 sequences was compiled and used to predict the evolutionary history of this gene lineage. Full nucleotide sequences were aligned using the online version of MAFFT[10] with a gap open penalty of 3.0, offset value of 0.8, and default parameters. To find the nucleotide substitution model that best fits our data, using the Akaike Information Criterion[11], we used jModelTest 2[12], which identified the GTRGAMMA model. RAxML v.8.0.0 was used to estimate phylogenetic relationships under a maximum likelihood (ML) framework[13]. The GTRGAMMA model was assigned, and a full ML search was implemented, using the autoMRE bootstrapping criterion to assess nodal support (-f a -# autoMRE option). Closely related genes from *Arabidopsis* - *ARF3* (*At2g33860*); *RAV1* (*At1g13260*); *RAV2* (*At1g25560*); *DREB1A* (*At4g25480*); *DRE1B* (*At4g25490*) - were used as the outgroup.

The *AP2/ERF* family is split into two major subclades, *euAP2* and *ANT* (*AINTEGUMENTA*), in which three and 11 *Ceratopteris* genes were placed, respectively (Supplementary Fig. 2). *CerAP2* was previously found to be expressed in the inner sporangium wall, young spores, and leaf vasculature during sporogenesis, based on *in situ* hybridization analyses[7]. We found *CerAP2* evenly expressed throughout the ten tissues, while the *Ceratopteris euANT* genes were more highly expressed in stem, root, and young sporophytes and the *Ceratopteris basalANT* genes were also evenly expressed throughout the different tissues (Supplementary Fig. 8).

Co-evolution of LEU- and SEU-like gene families

Publicly available (ncbi.nlm.nih.gov, fernbase.org, congenie.org) *LEUNIG-* (*LUG*) and *SEUSS-* (*SEU*) like genes of *Amborella trichopoda, Arabidopsis thaliana, Azolla filiculoides Chara braunii, Glycine max, Gnetum montanum, Klebsormidium nitens, Marchantia polymorpha, Nicotiana tabacum, Oryza sativa, Physcomitrium patens, Populus trichocarpa, Picea abies, Pinus pinaster, Pseudotsuga menziesii, Salvinia cucullata, Selaginella moellendorfii, Taxus baccata, Vitis vinifera, Volvox carteri f. niagaiensis* and *Zea mays* were identified using BLAST[14]. Phylogenetic trees were constructed using amino acid sequences aligned with MAFFT using MEGA X, maximum likelihood, and automatic trimming (60% site coverage)[15]. Expression patterns of *Ceratopteris LUG*-like and *SEU*-like genes were generated using Heatmapper[16].

LUG- and *SEU*-like genes encode for transcriptional co-regulators that interact and co-evolve even though they belong to different gene families. In flowering plants, these genes are essential for proper flower development, specifically in controlling petal shape and polarity. *LUG*-like genes of seed plants fall into two subclades, the *LEUNIG-HOMOLOG* (*LUH*) clade and the *LUG* clade, but all non-seed land plant sequences, including a single gene, *CrLUG,* from *Ceratopteris*, are members of the *LUG* clade only (Supplementary Fig. 9A). *SEU*-like genes of seed plants also form two clades, the *SEU* and *SEUSS*-like clades. Of the three *SEU*-like *Ceratopteris* genes, two clustered with the seed plant and bryophyte *SEU* genes, and one, *SEUSS*-like 3 (*CrSEU3*), appeared in a clade consisting only of fern sequences (Supplementary Fig. 9B). Neither the *LUG* nor the *SEU* gene families expanded or collapsed in *Ceratopteris* but both kept a moderate number of members during land plant evolution, suggesting that even after gene copies originating from a WGD are purged from the genome, the loss of one gene family member cannot be tolerated. *SEU*- and *LUG*-like *Ceratopteris* genes were expressed in sporophytic and gametophytic tissue alike, with lowest expression in sporangia and developing leaves, and highest expression in the stem, root, expanding leaf, and young sporophyte, suggesting pleiotropic functions for the members of both gene families.

Evolution and selection of cell cycle gene families

Homosporous plants have on average considerably higher chromosome numbers than do heterosporous plants[17,18]. Presumably, this is related either to mechanisms of genome doubling or of genome downsizing, and we hypothesize there could be differences in genes associated with the cell cycle in homosporous and heterosporous plants. Thus, we analyzed genes associated with cyclin-dependent kinases and cyclins, chromatin remodeling, and spindle formation and mapped significant gene copy expansions and contractions on a phylogenetic tree of land plants for which genome sequences are available (Supplementary Fig. 10).

We ran Orthofinder 2.4.0[19] on 21 plant species representing all major clades of land plants, for which whole genomes are currently available (July 2021). We selected genes associated with cyclin-dependent kinases and cyclins[20] and genes associated with spindle fiber formation and chromatin remodeling directly from The *Arabidopsis* Information Resource (TAIR), using the native search function in TAIR and the search terms “spindle” and “chromatin.” We selected 143 orthogroups from the output of Orthofinder and examined the evolution of gene copy number. For

this we used Computational Analysis of gene Family Evolution (CAFE) v5.0[21], which uses a birth and death model to simulate gene family evolution. We then mapped nodes and tips that CAFE indicated had significant expansions or retractions in copy number.

We aligned orthogroups with MUSCLE[22] and synchronized the headers of the peptide orthogroup files to match the corresponding CDS files (https://github.com/carol-rowe666/ortho_group_refile). We then used the peptide alignments to guide the CDS alignment with pal2nal (http://www.bork.embl.de/pal2nal/). Gene models in *Pinus taeda* were often mismatched between CDS and peptide sequences, so this species was removed from the analysis. The pal2nal-aligned CDS files were then used to generate gene trees using IQtree2 (https://github.com/iqtree/iqtree2). Three of the 129 orthogroups included fewer than four sequences and could not be bootstrapped for gene tree estimation. We paired the Newick outputs generated by IQtree2 with the pal2nal-generated aligned CDS orthogroups to test for selection using the absREL model built into Hyphy (http://hyphy.org).

We found no distinct patterns of changes in gene copy number in the three heterosporous lineages. However, in the lineage leading to *Ceratopteris*, we observed five gene expansion events: one associated with a cyclin and four associated with chromatin remodeling. Using HyPhy's implementation of the absREL model, one orthogroup with the largest number of *Ceratopteris* genes under selection included six genes undergoing positive selection. This orthogroup included *Arabidopsis* genes known to be involved in regulation of DNA repair, DNA binding, RNA binding, and endonuclease activity. Otherwise, most other orthogroups had no *Ceratopteris* genes with signs of positive selection and very few orthogroups with either one or two genes undergoing positive selection.

Cytochrome P450 gene family diversity across green plants

Cytochrome P450s (CYP450s) can be found across the major lineages of life but are most diverse in green plants. These enzymes are critical to plant metabolism and have diversified to become one of the largest metabolism-related enzyme families found in Viridiplantae[23]. We identified CYP450 genes from 40 green plant species with multiple representatives from each major lineage, plus three red and brown algae (Rhodophyta and Phaeophyceae, respectively) as outgroups, and classified them into previously identified CYP450 subfamilies (Supplementary Table 12). In total, we identified 11,463 CYP450 genes across these 43 species with the highest CYP450 diversity in hexaploid wheat, *Triticum aestivum*. On average, angiosperms contained 401 CYP450 genes, although this sampling does include recent polyploids such as *T. aestivum*. The gymnosperms had a mean count of 344 CYP450s, ferns had 181, the single lycophyte sample *Selaginella moellendorffii* had 308, bryophytes had 151, charophyte algae had 31, and chlorophyte algae had 17; within ferns, the two water ferns, *Azolla* and *Salvinia*, had 155 and 80 total CYP450 genes, respectively, and *Ceratopteris* had 309. The largest difference in CYP450 subfamily diversity between *Ceratopteris* and the water ferns was in CYP704B (58 in *Ceratopteris*, 7 in *Azolla*, 11 in *Salvinia*), which encode omega fatty acid hydroxylases, enzymes essential for spore

wall polymers[24]. Interestingly, this subfamily has even lower diversity in seed plants, with a mean count of 1.9 CYP704B genes.

Independent of the CYP450 subfamilies, which were largely identified from angiosperm reference genes, we constructed a CYP450 phylogeny from 14 of these taxa, with representatives from each major green plant lineage (Supplementary Figure 11). While the early-diverging CYP450 clades have representative genes from diverse green plant taxa, it is clear that lineage-specific subfamilies are prevalent especially in more recently diverged clades of the CYP450 genes.

Revitalizing the C-Fern Curriculum

Ceratopteris is an exemplary model for teaching plant development and genetics because it has both independent gametophytic and sporophytic life stages, clearly demonstrating the alternation of generations[25,26] typical of all land plants. Students can easily mutagenize the haploid spores using EMS or X-rays and see the resulting mutant phenotypes in the haploid gametophytes[27]. The ability of ferns to undergo intragametophytic selfing (egg fertilized by sperm from a single gametophyte) allows students to produce completely homozygous sporophytes in which any mutant phenotype will also be apparent in the diploid life cycle stage. In addition, true-breeding mutant inbred lines can be maintained long term or can easily be crossed with other genotypes. With the ease and low costs of sequencing, undergraduate students can identify mutant phenotypes in *Ceratopteris,* isolate RNA, prepare libraries, send the libraries off for sequencing, and ultimately receive training in how to map RNA-seq data onto a reference genome within a semester. *Ceratopteris* is also capable of undergoing apogamy (a haploid sporophyte forms from gametophytic tissue without fertilization) and apospory (diploid gametophytes form from sporophytic tissue), readily producing polyploid individuals and essentially permitting virtually every means of plant reproduction with this single species[28,29]. The genome assembly and the sequenced doubled haploid F_2 mapping population of *Ceratopteris* provide the resources and techniques to ensure that *Ceratopteris* and the C-Fern Curriculum will be an important model system for teaching plant development, genetics, genomics, breeding, physiology, and bioinformatics for years to come.

REFERENCES

1. Ma, L. Q. *et al.* A fern that hyperaccumulates arsenic. *Nature* **409**, 579 (2001).
2. Deng, F. *et al.* Metalloid hazards: from plant molecular evolution to mitigation strategies. *J. Hazard. Mater.* **409**, 124495 (2021).
3. Talebi, M., Tabatabaei, B. E. S. & Akbarzadeh, H. Hyperaccumulation of Cu, Zn, Ni, and Cd in Azolla species inducing expression of methallothionein and phytochelatin synthase genes. *Chemosphere* **230**, 488–497 (2019).
4. Praveen, A. & Pandey, V. C. Pteridophytes in phytoremediation. *Environ. Geochem. Health* 1–13 (2019).
5. Bora, M. S., Gogoi, N. & Sarma, K. P. Tolerance mechanism of cadmium in Ceratopteris pteridoides: Translocation and subcellular distribution. *Ecotoxicol. Environ. Saf.* **197**, 110599 (2020).
6. Thomine, S., Wang, R., Ward, J. M., Crawford, N. M. & Schroeder, J. I. Cadmium and iron transport by members of a plant metal transporter family in Arabidopsis with homology to Nramp genes. *Proc. Natl. Acad. Sci.* **97**, 4991–4996 (2000).
7. Zumajo-Cardona, C., Pabón-Mora, N. & Ambrose, B. A. The evolution of euAPETALA2 genes in vascular plants: from plesiomorphic roles in sporangia to acquired functions in ovules and fruits. *Mol. Biol. Evol.* **38**, 2319–2336 (2021).
8. Kim, S., Soltis, P. S., Wall, K. & Soltis, D. E. Phylogeny and Domain Evolution in the APETALA2-like Gene Family. *Mol. Biol. Evol.* **23**, 107–120 (2006).
9. Zumajo-Cardona, C. & Pabón-Mora, N. Evolution of the APETALA2 gene lineage in seed plants. *Mol. Biol. Evol.* **33**, 1818–1832 (2016).
10. Katoh, K. & Standley, D. M. MAFFT multiple sequence alignment software version 7: improvements in performance and usability. *Mol. Biol. Evol.* **30**, 772–780 (2013).
11. Akaike, H. Stochastic theory of minimal realization. *IEEE Trans. Automat. Contr.* **19**, 667–674 (1974).
12. Darriba, D., Taboada, G. L., Doallo, R. & Posada, D. jModelTest 2: more models, new heuristics and parallel computing. *Nat. Methods* **9**, 772 (2012).
13. Stamatakis, A. RAxML version 8: a tool for phylogenetic analysis and post-analysis of large phylogenies. *Bioinforma.* **30**, 1312–1313 (2014).
14. Altschul, S. *et al.* Gapped BLAST and PSI-BLAST: a new generation of protein database search programs. *Nucleic Acids Res* **25**, (1997).
15. Kumar, S., Stecher, G., Li, M., Knyaz, C. & Tamura, K. MEGA X: molecular evolutionary genetics analysis across computing platforms. *Mol. Biol. Evol.* **35**, 1547 (2018).
16. Babicki, S. *et al.* Heatmapper: web-enabled heat mapping for all. *Nucleic Acids Res.* **44**, W147–W153 (2016).
17. Klekowski, E. & Baker, H. Evolutionary Significance of Polyploidy in the Pteridophyta. *Science* **153**, 305–307 (1966).
18. Sessa, E. B. & Der, J. P. Evolutionary genomics of ferns and lycophytes. in *Advances in Botanical Research* **78**, 215–254 (Elsevier, 2016).
19. Emms, D. M. & Kelly, S. OrthoFinder: solving fundamental biases in whole genome comparisons dramatically improves orthogroup inference accuracy. *Genome Biol.* **16**, 157 (2015).
20. Vandepoele, K. *et al.* Genome-wide analysis of core cell cycle genes in Arabidopsis. *Plant Cell* **14**, 903–916 (2002).

21. De Bie, T., Cristianini, N., Demuth, J. P. & Hahn, M. W. CAFE: a computational tool for the study of gene family evolution. *Bioinformatics* **22**, 1269–1271 (2006).
22. Edgar, R. C. MUSCLE: a multiple sequence alignment method with reduced time and space complexity. *BMC Bioinformatics* **5**, 1–19 (2004).
23. Nelson, D. & Werck-Reichhart, D. A P450-centric view of plant evolution. *Plant J.* **66**, 194–211 (2011).
24. Morant, M. *et al.* CYP703 is an ancient cytochrome P450 in land plants catalyzing in-chain hydroxylation of lauric acid to provide building blocks for sporopollenin synthesis in pollen. *Plant Cell* **19**, 1473–1487 (2007).
25. Spiro, M. D. & Knisely, K. I. Alternation of generations and experimental design: a guided-inquiry lab exploring the nature of the her1 developmental mutant of Ceratopteris richardii (C-Fern). *CBE—Life Sci. Educ.* **7**, 82–88 (2008).
26. Hickok, L. G., Warne, T. R., Baxter, S. L. & Melear, C. T. Education: Sex and the C-Fern: Not just another life cycle. *Bioscience* **48**, 1031–1037 (1998).
27. Hickok, L. G., Warne, T. R. & Slocum, M. K. Ceratopteris richardii: applications for experimental plant biology. *Am. J. Bot.* **74**, 1304–1316 (1987).
28. Hickok, L. G. Apogamy and somatic restitution in the fern Ceratopteris. *Am. J. Bot.* **66**, 1074–1078 (1979).
29. DeYoung, B., Weber, T., Hass, B. & Banks, J. A. Generating Autotetraploid Sporophytes and Their Use in Analyzing Mutations Affecting Gametophyte Development in the Fern Ceratopteris. *Genetics* **147**, 809 LP – 814 (1997).

SUPPLEMENTARY FIGURES

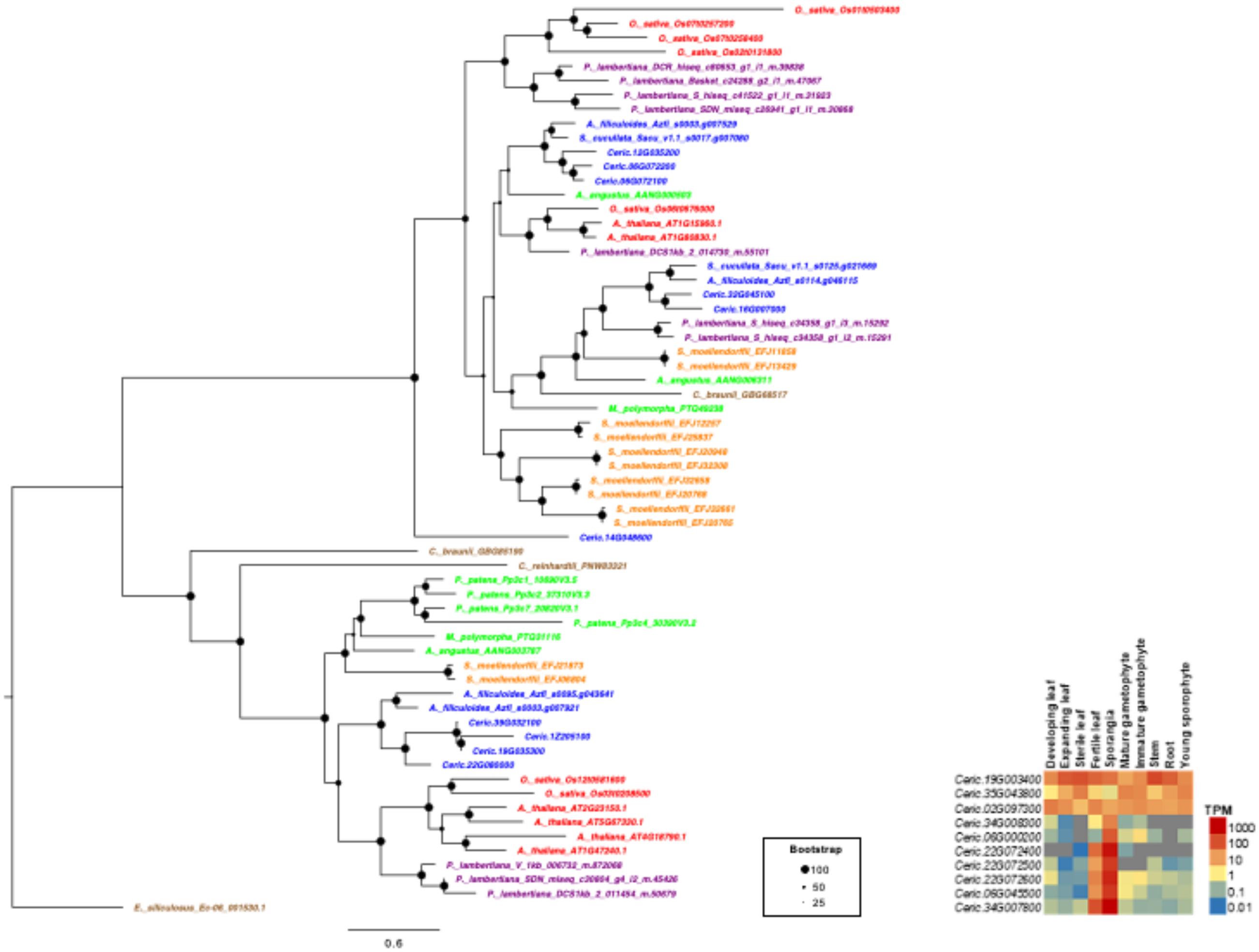

Supplementary Fig. 1 Phylogeny of *NRAMP* metal-ion transporter gene family and *Ceratopteris NRAMP* gene expression. Angiosperm genes are red, gymnosperm genes are purple, fern genes are blue, lycophyte genes are orange, bryophyte genes are green, and algal genes are brown.

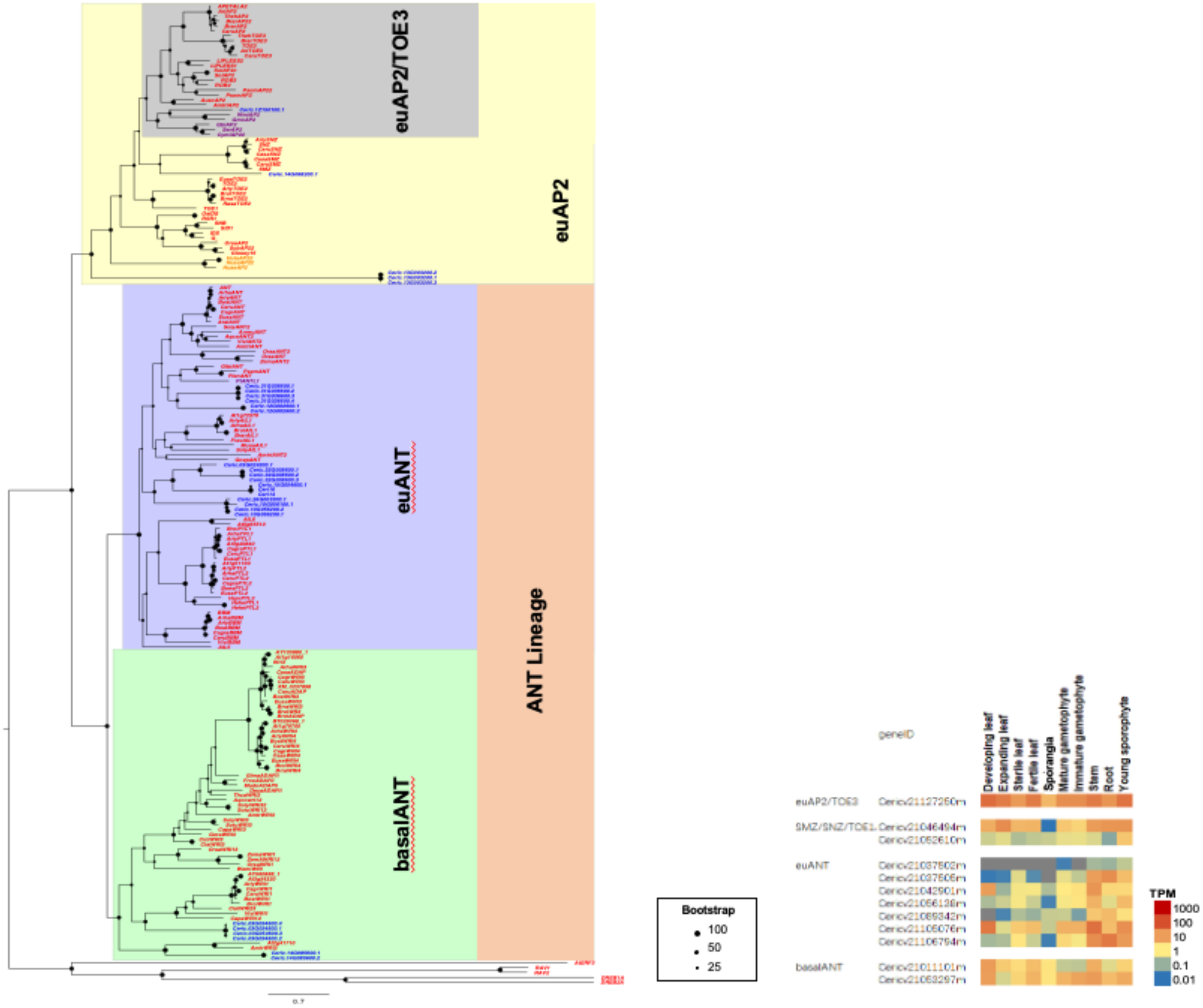

Supplementary Fig. 2 Phylogeny of the *APETALA2/ETHYLENE RESPONSIVE FACTOR* (*AP2/ERF*) across land plants and expression in *Ceratopteris*. Angiosperm genes are red, gymnosperm genes are purple, fern genes are blue, and lycophyte genes are orange.

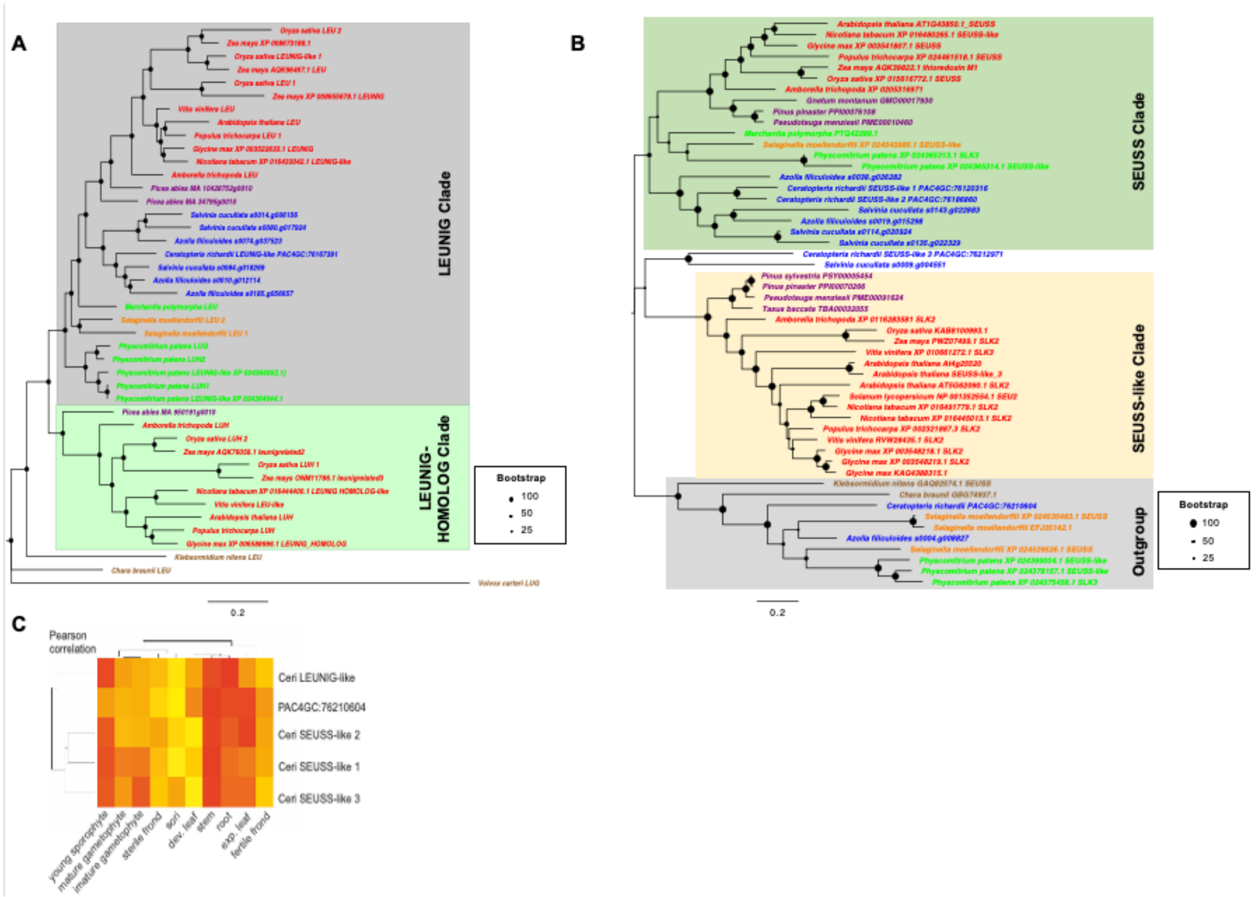

Supplementary Fig. 3 Co-evolution of transcriptional regulators. (A) Phylogeny of *LEUNIG*-like gene family. (B) Phylogeny of *SEUSS*-like gene family. Angiosperm genes are red, gymnosperm genes are purple, fern genes are blue, lycophyte genes are orange, bryophyte genes are green, and algal genes are brown. (C) Gene expression patterns of *LEU*- and *SEU*-like genes in tissues/life stages of *Ceratopteris*.

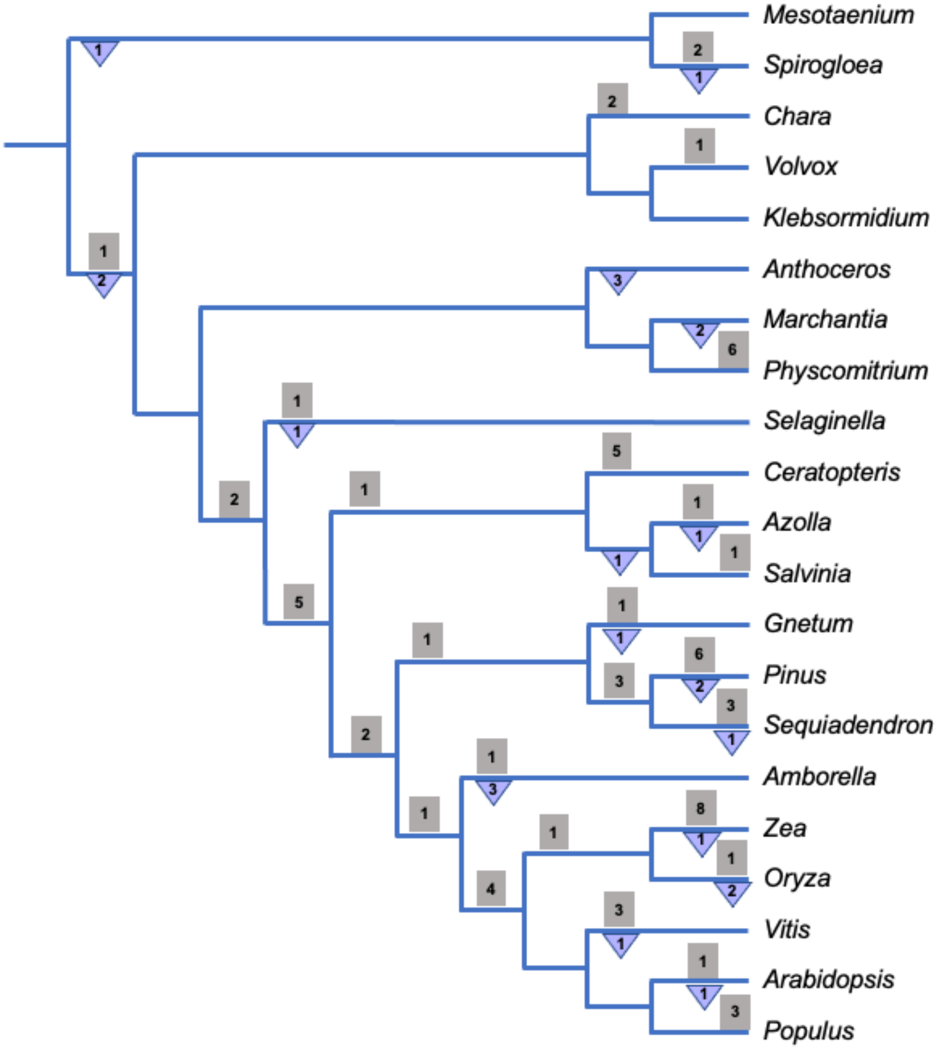

Supplementary Fig. 4 Expansion and contraction in copy number of cell cycle-related genes across green plants. Light purple triangles indicate contractions; grey squares show expansions.

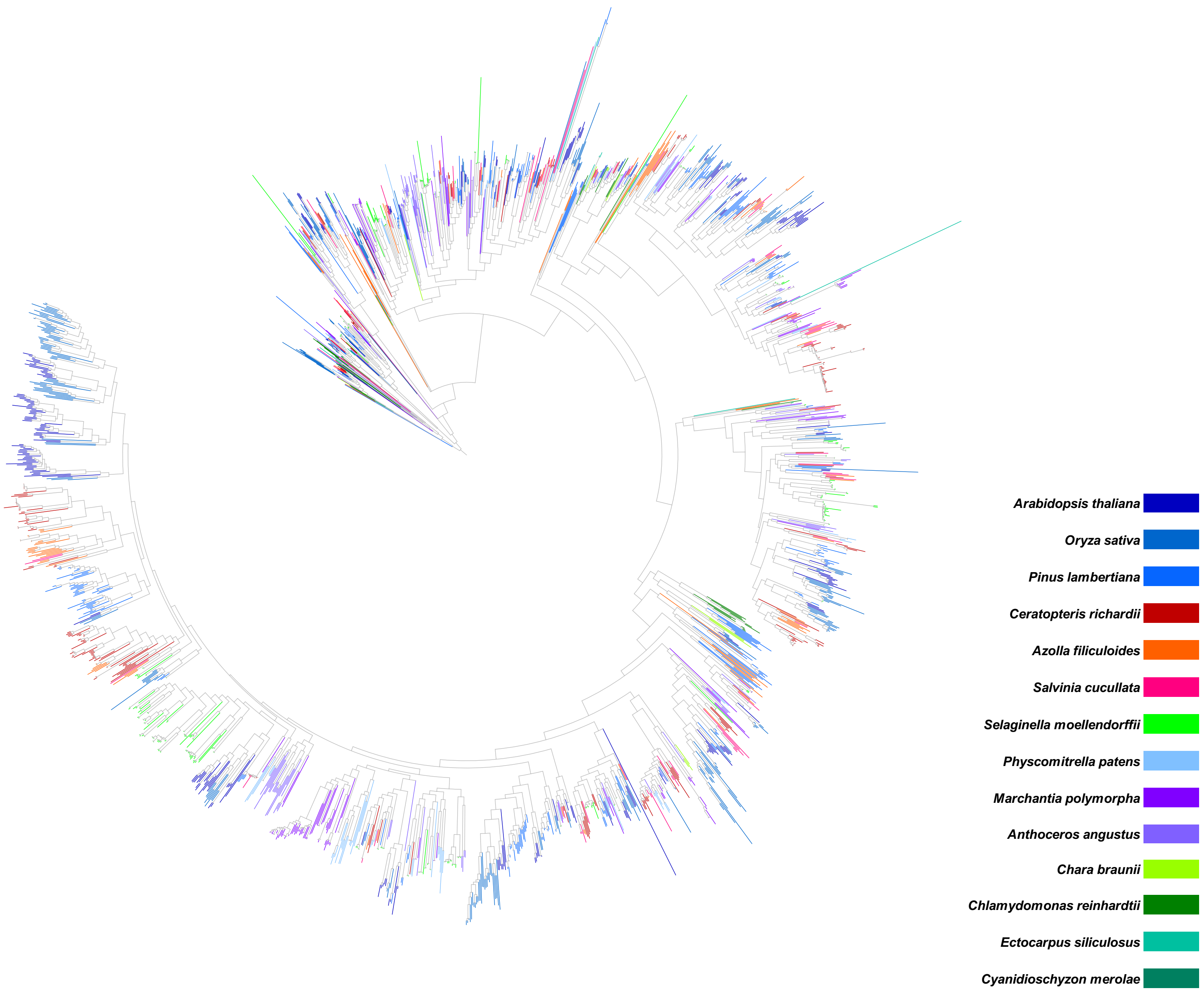

Supplementary Fig. 5 CYP450 gene diversity and evolution across green plants. The color-coding of the 14 green plant taxa spaning 2,193 genes is found in the figure legend.
