# Supplementary material for: Dynamic genome evolution in a model fern
Source: Nat Plants. 2022 Sep 1;8(9):1038–51. doi: 10.1038/s41477-022-01226-7 (PMC9477723; doi:10.1038/s41477-022-01226-7)
Supplement: Supplementary file 2 — Reporting Summary [file 41477_2022_1226_MOESM2_ESM.pdf]

Corresponding author(s): D. Blaine Marchant, James H. Leebens-Mack, Pamela S. Soltis, Douglas E. Soltis, Zhong-Hua Chen

Last updated by author(s): Jul 7, 2022

Reporting Summary

Nature Research wishes to improve the reproducibility of the work that we publish. This form provides structure for consistency and transparency in reporting. For further information on Nature Research policies, see our Editorial Policies and the Editorial Policy Checklist.

Statistics

For all statistical analyses, confirm that the following items are present in the figure legend, table legend, main text, or Methods section.

n/a	Confirmed	
☐	☒	The exact sample size (*n*) for each experimental group/condition, given as a discrete number and unit of measurement
☒	☐	A statement on whether measurements were taken from distinct samples or whether the same sample was measured repeatedly
☒	☐	The statistical test(s) used AND whether they are one- or two-sided<br>*Only common tests should be described solely by name; describe more complex techniques in the Methods section.*
☒	☐	A description of all covariates tested
☐	☒	A description of any assumptions or corrections, such as tests of normality and adjustment for multiple comparisons
☐	☒	A full description of the statistical parameters including central tendency (e.g. means) or other basic estimates (e.g. regression coefficient) AND variation (e.g. standard deviation) or associated estimates of uncertainty (e.g. confidence intervals)
☐	☒	For null hypothesis testing, the test statistic (e.g. *F*, *t*, *r*) with confidence intervals, effect sizes, degrees of freedom and *P* value noted<br>*Give P values as exact values whenever suitable.*
☒	☐	For Bayesian analysis, information on the choice of priors and Markov chain Monte Carlo settings
☒	☐	For hierarchical and complex designs, identification of the appropriate level for tests and full reporting of outcomes
☒	☐	Estimates of effect sizes (e.g. Cohen's *d*, Pearson's *r*), indicating how they were calculated

Our web collection on statistics for biologists contains articles on many of the points above.

Software and code

Policy information about availability of computer code

Data collection	Illumina and PacBio reads were sequenced at the Department of Energy (DOE) Joint Genome Institute (JGI) in Walnut Creek, California, USA, and the HudsonAlpha Institute for Biotechnology in Huntsville, Alabama, USA. Illumina reads were sequenced using the Illumina NovaSeq platform, and the PacBio reads were sequenced using the SEQUEL platform.
Data analysis	MECAT assembler v1.1 (Xiao et al., 2017)<br>Canu assembler v1.8 (Koren et al., 2017)<br>ARROW (Chin et al., 2013)<br>JUICER (Durand et al., 2016)<br>bwa mem (Li, 2013)<br>GATK (McKenna et al., 2010)<br>PERTRAN GSNAP (Wu and Nacu, 2010)<br>PASA (Haas et al., 2003)<br>EXONERATE (Slater and Birney, 2005)<br>RepeatMasker (Smit et al., 2013)<br>RepeatModeler (Smit and Hubley, 2008)<br>FGENESH+ (Salamov and Solovyev, 2000)<br>BLAT (Kent, 2002)<br>HISAT2 (Kim et al., 2019) v2.2.1<br>samtools v1.11 (Li et al., 2009)<br>Stringtie v2.1.4 (Pertea et al., 2015)<br>ballgown v2.20.0 (Frazee et al., 2015)<br>clusterProfiler v3.16.1 (Yu et al., 2012)<br>Vennerable v3.0 (https://R-Forge.R-project.org/projects/vennerable/)<br>pheatmap v1.0.12 (https://CRAN.R-project.org/package=pheatmap)

GENESPACE (https://code.jgi.doe.gov/plant/genespace-r)(Lovell et al., 2018)
MCScanX (Wang et al., 2012)
Orthofinder 2.4.0 (Emms and Kelly, 2015)
Wgd (Zwaenepoel and Van de Peer, 2019)
BLASTp (Camacho et al., 2009)
MCL (Altschul et al., 1997)
PAML (Yang, 2007)
ggplot2 v3.3.3(Wickham, 2016)
PASTA (Mirarab et al., 2015)
WGDgc (Rabier et al., 2014)
GenPhyloData (Sjöstrand et al., 2013)
NOTUNG v. 2.9.1.5 (Stolzer et al., 2012)
MAPS (Li et al., 2015)
RAxML v. 8.2.11 (Stamatakis, 2014)
Frackify (https://gitlab.com/barker-lab/frackify)
MASS R (Venables and Ripley, 2002)
Methylpy v. 1.4.2 (Schultz et al., 2015)
cutadapt v1.18 (Martin, 2011)
bowtie v2.4.1 (Langmead and Salzberg, 2012)
MAFFT (Katoh and Standley, 2013)
IQ-Tree (Minh et al., 2020)
HMMER 3.1b2 (hmmer.org)
AUGUSTUS 3.3.2 (Stanke et al., 2006)
BLAST+ (Altschul et al., 1990)
Probalign (Roshan and Livesay, 2006)
trimAl 1.2rev59t (Capella-Gutiérrez et al., 2009)
jModelTest 2 (Darriba et al., 2012)
Heatmapper (Babicki et al., 2016)
CAFE v5.0 (De Bie et al., 2006)
MUSCLE (Edgar, 2004)
pal2nal (http://www.bork.embl.de/pal2nal/)
Hyphy (http://hyphy.org)

For manuscripts utilizing custom algorithms or software that are central to the research but not yet described in published literature, software must be made available to editors and reviewers. We strongly encourage code deposition in a community repository (e.g. GitHub). See the Nature Research guidelines for submitting code & software for further information.

Data

Policy information about availability of data

All manuscripts must include a data availability statement. This statement should provide the following information, where applicable:

- Accession codes, unique identifiers, or web links for publicly available datasets
- A list of figures that have associated raw data
- A description of any restrictions on data availability

Raw genomic sequences have been deposited in the NCBI SRA under BioProject PRJNA729743. Genome and transcriptome assemblies and annotations can be found in Phytozome (https://phytozome-next.jgi.doe.gov/info/Crichardii_v2_1). Publicly available data was collected from Ensembl Plants (plants.ensembl.org), NCBI (ncbi.nlm.nih.gov), Swiss-Prot (uniprot.org), RepBase (girinst.org/repbase), One Thousand Plant Transcriptomes (1KP) database (OTPT Initiative, 2019), fern transcriptome database (Shen et al., 2018), water fern genomes (fernbase.org), spruce genome (congenie.org), TAIR (arabidopsis.org).

Field-specific reporting

Please select the one below that is the best fit for your research. If you are not sure, read the appropriate sections before making your selection.

☒ Life sciences ☐ Behavioural & social sciences ☐ Ecological, evolutionary & environmental sciences

For a reference copy of the document with all sections, see nature.com/documents/nr-reporting-summary-flat.pdf

Life sciences study design

All studies must disclose on these points even when the disclosure is negative.

Sample size	For phylogenetic analyses, bootstrapping values were set to 100-1000, as per the norm in phylogenetics. For gene feature/genome characteristics (intron length, exon length, methylation levels, etc) analyses all features from the genome were incorporated unless otherwise noted. For MAPS, 3000 gene trees were simulated for each species tree with at least one tip per species: 1000 gene trees at the estimated λ and μ, 1000 gene trees at half of the estimated λ and μ, and 1000 trees at three times λ and μ.
Data exclusions	No data were excluded from the analyses.
Replication	Four biological replicates were used for each tissue sampled for RNA-seq. Six biological replicates were used for the metabolite analyses.
Randomization	The genomic, transcriptomic, methylomic, and metabolomic data were all generated from the Hn-n genotype of Ceratopteris richardii. or

Randomization	gene feature/genome characteristics (intron length, exon length, methylation levels, etc) analyses all features from the genome were incorporated unless otherwise noted.
Blinding	No blinding is necessary for genome assembly, characterization, comparisons, or evolutionary analyses.

Reporting for specific materials, systems and methods

We require information from authors about some types of materials, experimental systems and methods used in many studies. Here, indicate whether each material, system or method listed is relevant to your study. If you are not sure if a list item applies to your research, read the appropriate section before selecting a response.

Materials & experimental systems

n/a	Involved in the study
☒	☐ Antibodies
☒	☐ Eukaryotic cell lines
☒	☐ Palaeontology and archaeology
☒	☐ Animals and other organisms
☒	☐ Human research participants
☒	☐ Clinical data
☒	☐ Dual use research of concern

Methods

n/a	Involved in the study
☒	☐ ChIP-seq
☒	☐ Flow cytometry
☒	☐ MRI-based neuroimaging
